# Prognostic and predictive value of a pathomics signature in gastric cancer

**DOI:** 10.1038/s41467-022-34703-w

**Published:** 2022-11-12

**Authors:** Dexin Chen, Meiting Fu, Liangjie Chi, Liyan Lin, Jiaxin Cheng, Weisong Xue, Chenyan Long, Wei Jiang, Xiaoyu Dong, Jian Sui, Dajia Lin, Jianping Lu, Shuangmu Zhuo, Side Liu, Guoxin Li, Gang Chen, Jun Yan

**Affiliations:** 1 Department of General Surgery, Guangdong Provincial Key Laboratory of Precision Medicine for Gastrointestinal Tumor, Nanfang Hospital, The First School of Clinical Medicine, Southern Medical University, 510515 Guangzhou, P.R. China; 2 School of Science, Jimei University, 361021 Xiamen, P.R. China; 3 Department of Gastroenterology, Guangdong Provincial Key Laboratory of Gastroenterology, Nanfang Hospital, The First School of Clinical Medicine, Southern Medical University, 510515 Guangzhou, P.R. China; 4 Department of Gastrointestinal Surgery, Fujian Provincial Hospital, Teaching Hospital of Fujian Medical University, 350001 Fuzhou, P.R. China; 5 Department of Pathology, Fujian Key Laboratory of Translational Cancer Medicine, Clinical Oncology School of Fujian Medical University, Fujian Cancer Hospital, 350014 Fuzhou, P.R. China

**Keywords:** Gastric cancer, Prognostic markers

## Abstract

The current tumour-node-metastasis (TNM) staging system alone cannot provide adequate information for prognosis and adjuvant chemotherapy benefits in patients with gastric cancer (GC). Pathomics, which is based on the development of digital pathology, is an emerging field that might improve clinical management. Herein, we propose a pathomics signature (PS_GC_) that is derived from multiple pathomics features of haematoxylin and eosin-stained slides. We find that the PS_GC_ is an independent predictor of prognosis. A nomogram incorporating the PS_GC_ and TNM staging system shows significantly improved accuracy in predicting the prognosis compared to the TNM staging system alone. Moreover, in stage II and III GC patients with a low PS_GC_ (but not in those with a high PS_GC_), satisfactory chemotherapy benefits are observed. Therefore, the PS_GC_ could serve as a prognostic predictor in patients with GC and might be a potential predictive indicator for decision-making regarding adjuvant chemotherapy.

## Abstract (web summary)

The current tumour-node-metastasis staging system alone cannot provide adequate information for prognosis and adjuvant chemotherapy benefits in patients with gastric cancer. Herein, the authors propose a pathomics signature that was derived from multiple features of haematoxylin and eosin-stained slides.

## Introduction

Despite the remarkably reduced incidence and mortality of gastric cancer (GC), it remains an important contributor to the global burden of cancer^1^. Currently, the tumour-node-metastasis (TNM) staging system is considered the cornerstone for prognosis prediction and treatment decision-making in GC^2^. However, prognostic stratification of patients with GC according to the latest TNM staging system is often poor^3^. Adjuvant chemotherapy is recommended for advanced GC because of the improvement of oncological outcomes, but large variations in survival benefits from adjuvant chemotherapy have been reported even in patients with the same stage of disease and receiving similar treatment regimens^3,4^. These findings suggest that the present TNM staging system provides inadequate prognostic information and cannot accurately identify patients who are more likely to benefit from adjuvant chemotherapy, which highlights the urgent need for discovering new biomarkers that are associated with prognosis and adjuvant chemotherapy benefits in GC.

To understand the heterogeneous prognoses and adjuvant chemotherapy benefits seen in the clinic, several subtyping algorithms based on gene expression data have been investigated^5–7^. Although these methods have greatly improved our knowledge regarding GC and the potential of subgroup-specific rational treatment strategies has been enumerated by several studies, the cost and complexity of transcriptomic analyses, including expression microarray and RNA-seq analyses, prevent their active utilization in clinical practice^8,9^.

Evaluation of haematoxylin and eosin (H&E)-stained slides by experienced pathologists is indispensable for determining the TNM stage and histological classification of GC cases in the clinic. Full digitalization of the stained tissue sections has become feasible because of advances in slide scanning technology and reductions in the cost of digital storage^10^. Recently, the term “pathomics” has attracted increased attention. Pathomics embodies a wide variety of data that are captured from digital pathology image analyses to generate quantitative features for characterizing diverse phenotypes of tissue samples, and these data are subsequently analysed to determine diagnosis or predict survival outcomes^11–13^. Therefore, we hypothesized that analyses of the automatic digital pathomics features extracted from H&E-stained slides could predict the prognosis and survival benefits associated with adjuvant chemotherapy in patients with GC.

Integration of multiple features into a single signature, rather than individual analyses, might improve the performance of the prognostic prediction^14,15^. The least absolute shrinkage and selection operator (LASSO)-Cox regression model is a state-of-the-art machine learning method for regression analysis of the relationships between high-dimensional features and survival^16–18^. Here, we propose a pathomics signature of GC (PS_GC_) that was developed with multiple pathomics features extracted from H&E-stained sections using a LASSO-Cox regression model. Thus, in this study, we intended to assess the prognostic value of the PS_GC_ for overall survival (OS) and disease-free survival (DFS) and explore whether the PS_GC_ could identify patients with stage II and III diseases who might benefit from adjuvant chemotherapy.

## Results

### Participants

Table 1 lists the clinicopathological characteristics of patients in the training (*n* = 264) and validation (*n* = 216) cohorts. Of the 480 patients included in this study, 69.4% (333/480) were male, and the median [interquartile range (IQR)] age was 58 (49–65) years. The majority of the patients (76.3%, 366/480) were diagnosed with stage II or III disease. No significant difference in clinicopathological characteristics between the training and validation cohorts was found. The clinicopathological characteristics of patients with and without complete data were similar (Supplementary Table 1). The median (IQR) follow-up duration in the training cohort was 64 (27–72) months, with 5-year OS and DFS rates of 58.7% and 55.3%, respectively (Supplementary Fig. 1a, b). In the validation cohort, the median (IQR) follow-up duration was 55 (22.25–92) months. The 5-year OS and DFS rates were 47.7% and 45.4%, respectively (Supplementary Fig. 1c, d).

**Table 1:** Characteristics of patients in the training and validation cohorts

Variables	Training cohort (*n* = 264)		Validation cohort (*n* = 216)		*P*
	*n*	%	*n*	%	
**Age**					0.417
≤60 years	160	60.6	123	56.9	
>60 years	104	39.4	93	43.1	
**Age (years), median (IQR)**	57 (49–65)		59 (51–65)		0.443
**Sex**					0.571
Male	186	70.5	147	68.1	
Female	78	29.5	69	31.9	
**ECOG PS**					0.840
0	190	72.0	158	73.1	
1	68	25.8	55	25.5	
2	6	2.2	3	1.4	
**CEA level**					0.158
Normal	216	81.8	187	86.6	
Elevated	48	18.2	29	13.4	
**CA 19-9 level**					0.385
Normal	223	84.5	176	81.5	
Elevated	41	15.5	40	18.5	
**Tumour location**					0.690
Fundus of the stomach	61	23.1	43	19.9	
Body of the stomach	49	18.6	43	19.9	
Antrum of the stomach	154	58.3	130	60.2	
**Tumour size**					0.299
≤4 cm	147	55.7	110	50.9	
>4 cm	117	44.3	106	49.1	
**Tumour grade**					0.725
Grade 1	18	6.8	12	5.6	
Grade 2	54	20.5	44	20.4	
Grade 3	169	64.0	135	62.5	
Grade 4	23	8.7	25	11.5	
**Lauren type**					0.530
Intestinal type	120	45.5	92	42.6	
Diffuse and mixed type	144	54.5	124	57.4	
**Depth of invasion**					0.307
T1	48	18.2	30	13.9	
T2	24	9.1	17	7.9	
T3	29	11.0	19	8.8	
T4a	143	54.2	124	57.4	
T4b	20	7.5	26	12.0	
**Lymph node metastasis**					0.803
N0	112	42.4	81	37.5	
N1	50	18.9	40	18.5	
N2	42	15.9	38	17.6	
N3a	37	14.0	34	15.7	
N3b	23	8.8	23	10.7	
**Distant metastasis**					0.999
M0	253	95.8	207	95.8	
M1	11	4.2	9	4.2	
**TNM stage**					0.310
Stage I	54	20.5	30	13.9	
Stage II	74	28.0	66	30.5	
Stage III	125	47.3	111	51.4	
Stage IV	11	4.2	9	4.2	
**Adjuvant chemotherapy**					0.626
Yes	161	61.0	127	58.8	
No	103	39.0	89	41.2	

The comparisons of continuous age variables between two groups are performed using a two-sided Mann–Whitney *U* test, and the rest variables are compared using a two-sided *χ*^2^ or Fisher’s exact test.

*IQR* interquartile range, *ECOG PS* Eastern Cooperative Oncology Group performance status, *CEA* carcinoembryonic antigen, *CA* carbohydrate antigen, *TNM* tumour-node-metastasis.

### Construction of the PS_GC_

**Fig. 1:**
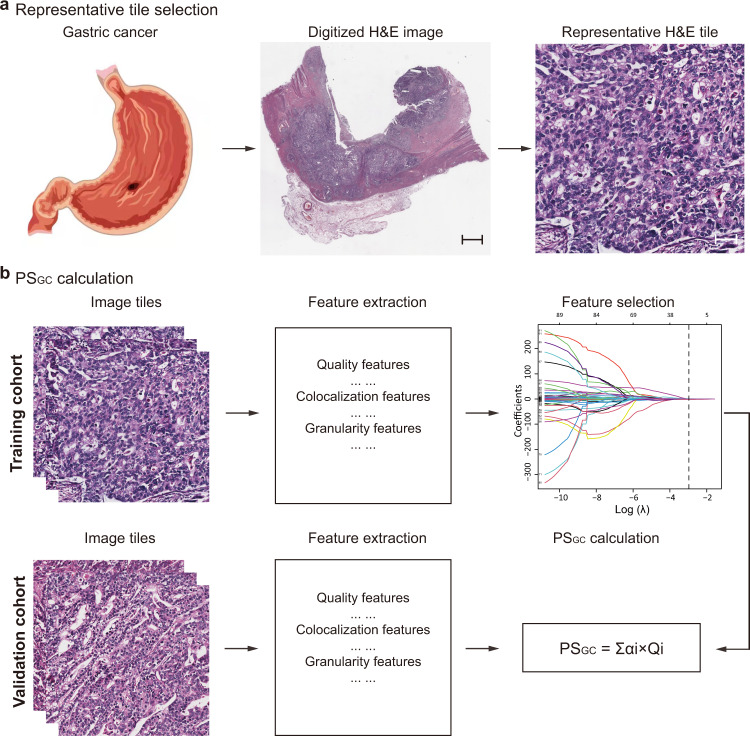
Schematic illustration of PS_GC_ construction. **a** Selection of a representative H&E tile. The H&E-stained images of all included 480 patients are used, and 10 regions of interest with a field of view of 1000 × 1000 pixels per image containing the greatest number of tumour cells are randomly selected for analysis. One pixel is equal to 0.504 μm. Scale bars: 1500 and 50 μm, respectively. **b** Framework for constructing the PS_GC_. The pathomics features are extracted from the H&E-stained image tiles, and then the potential predictors are selected using a LASSO-Cox regression model in the training cohort with 264 patients. The PS_GC_ is calculated via a linear combination of the selected features, and the PS_GC_ for the validation cohort with 216 patients is directly calculated from the formula obtained in the training cohort. PS_GC_ pathomics signature of gastric cancer, H&E haematoxylin and eosin, LASSO least absolute shrinkage and selection operator.

The framework for constructing the PS_GC_ is presented in Fig. 1. In the training cohort, a LASSO-Cox regression model with 10-fold cross-validation was used to construct the PS_GC_. The final PS_GC_ included 12 pathomics features (Supplementary Fig. 2). The PS_GC_ calculation formula is presented in the Supplementary Note, from which the PS_GC_ of the validation cohort was acquired directly. No statistically significant difference in the distribution of the PS_GC_ [median (IQR)] was found between the training [1.211 (0.857–1.593)] and validation [1.196 (0.902–1.734)] cohorts [median difference: −0.050; 95% confidence interval (CI): −0.151 to 0.051; *P* = 0.340]. In particular, compared with the intestinal type, a significantly higher PS_GC_ was detected in diffused and mixed type, with a median difference of 0.376 (95% CI: 0.250–0.497; *P* < 0.001) in the training cohort and 0.393 (95% CI: 0.258–0.541; *P* < 0.001) in the validation cohort (Supplementary Fig. 3). In terms of the tumour grade, the PS_GC_ in patients with grade 3 and grade 4 tumours was significantly higher than that in patients with grade 1 and grade 2 tumours in both the training (median difference: 0.415; 95% CI: 0.271–0.553; *P* < 0.001) and validation (median difference: 0.463; 95% CI: 0.307–0.630; *P* < 0.001) cohorts (Supplementary Fig. 4). In addition, the distribution of PS_GC_ was similar according to the tumour size subgroups in both the training (median difference: 0.099; 95% CI: −0.036 to 0.235; *P* = 0.158) and validation (median difference: 0.119; 95% CI: −0.031 to 0.268; *P* = 0.125) cohorts (Supplementary Fig. 5).

### Association of the PS_GC_ with prognosis

An optimum cutoff value of 1.16, which provided the highest standardized log-rank statistic, was determined with the training cohort (Supplementary Fig. 6). Accordingly, patients in both the training and validation cohorts were classified into high- and low-PS_GC_ groups. The distribution of the PS_GC_ across survival statuses as well as select pathomics features is shown in Supplementary Fig. 7, which revealed that a higher PS_GC_ was associated with a higher risk of recurrence or death.

**Fig. 2:**
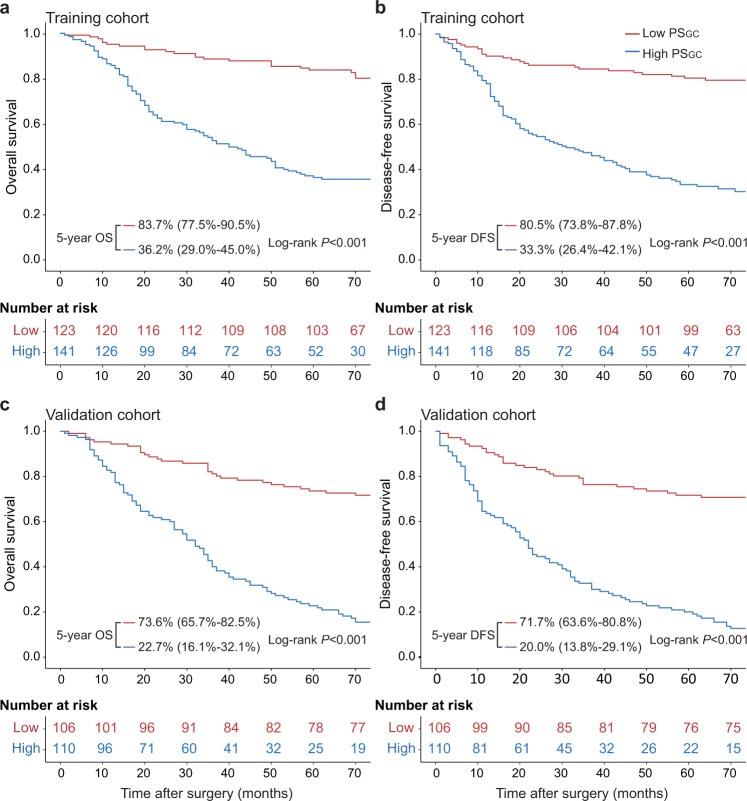
Kaplan–Meier survival curves according to the PS_GC_ level. **a** The OS rate difference between the high- and low-PS_GC_ patients in the training cohort. **b** The DFS rate difference between the high- and low-PS_GC_ patients in the training cohort. **c** The OS rate difference between the high- and low-PS_GC_ patients in the validation cohort. **d** The DFS rate difference between the high- and low-PS_GC_ patients in the validation cohort. The comparisons of OS and DFS between the two groups are performed using a two-sided log-rank test. OS overall survival, DFS disease-free survival, PS_GC_ pathomics signature of gastric cancer. Source data are provided as a Source data file.

In the training cohort, the 5-year OS and DFS rates were 83.7% and 80.5% in low-PS_GC_ patients, respectively, which were significantly reduced to 36.2% and 33.3% in high-PS_GC_ patients (Fig. 2a, b, both log-rank *P* < 0.001). We subsequently performed the same analyses in the validation cohort. Among low-PS_GC_ patients, the 5-year OS and DFS rates were 73.6% and 71.7%, respectively, and significantly worse 5-year OS and DFS rates of 22.7% and 20.0% were found in high-PS_GC_ patients (Fig. 2c, d, both log-rank *P* < 0.001). The PS_GC_ remained a significant prognostic indicator after stratification by clinicopathological variables, indicating the independent association of the PS_GC_ with the prognosis (Supplementary Figs. 8–11).

### Development and validation of the pathomics nomogram for prognosis

**Fig. 3:**
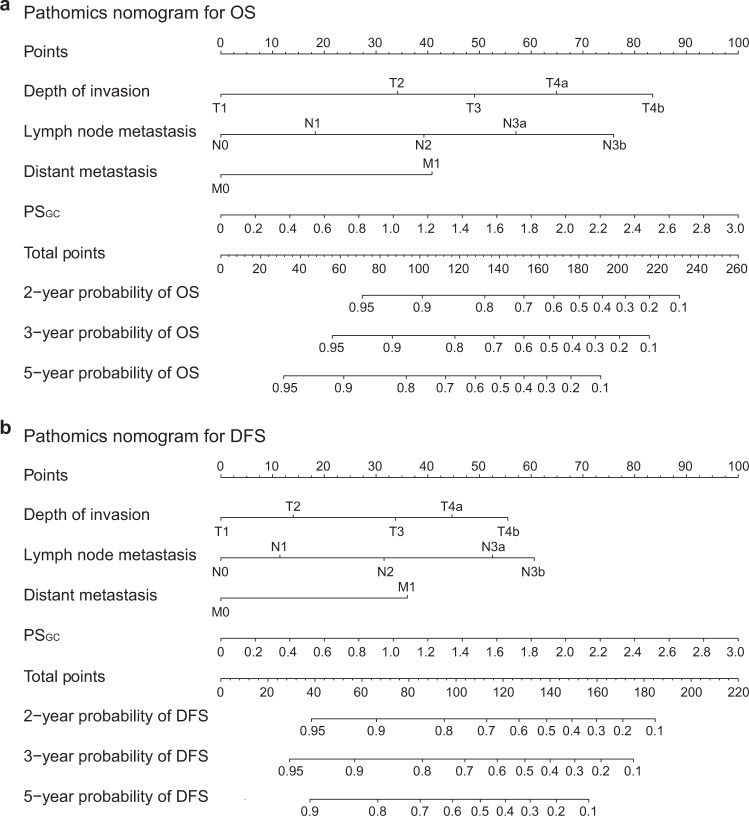
Pathomics nomograms for the prediction of OS and DFS. **a** Pathomics nomogram for OS. **b** Pathomics nomogram for DFS. The patient’s T stage on the depth of the invasion axis is first located using the nomogram. Then, a line is drawn straight upward to the Points axis to determine how many points the patient receives from the T stage. This process is repeated for each variable, and the points obtained from each risk factor are summed. Finally, the final sum is located on the Total point axis. A line is drawn straight down to find the patient’s probability of survival. OS overall survival, DFS disease-free survival, PS_GC_ pathomics signature of gastric cancer. Source data are provided as a Source data file.

In the univariate Cox regression analysis, the PS_GC_, carcinoembryonic antigen (CEA) level, carbohydrate antigen (CA) 19-9 level, tumour location, tumour size, Lauren type, depth of invasion (T stage), lymph node metastasis (N stage) and distant metastasis (M stage) were significantly associated with OS in the training cohort (Table 2). The backwards stepwise multivariate Cox regression analysis showed that the PS_GC_, depth of invasion, lymph node metastasis and distant metastasis were independent predictors of OS. The same results were found in the Cox regression analysis for DFS. The proportional hazards (PH) assumption tests for the Cox regression models were valid (Supplementary Figs. 12 and 13). No interaction effects were observed between PS_GC_ and the TNM staging system for OS and DFS (Supplementary Tables 2–4). Therefore, two pathomics nomograms were developed to predict OS and DFS by incorporating the four independent predictors (Fig. 3). Lymph node metastasis had the most important contribution to the prognostic prediction in the pathomics nomograms, followed by the PS_GC_ (Supplementary Fig. 14).

**Table 2:** Univariate and multivariate Cox regression analyses of the PS_GC_ and clinicopathological characteristics for overall survival and disease-free survival in training cohort

Variables	Overall survival				Disease-free survival			
	Univariate analysis		Multivariate analysis		Univariate analysis		Multivariate analysis	
	HR (95% CI)	*P*	HR (95% CI)	*P*	HR (95% CI)	*P*	HR (95% CI)	*P*
**Age** (years) (>60 years vs. ≤60 years)	1.153 (0.794–1.675)	0.454			1.107 (0.771–1.590)	0.583		
**Sex** (female vs. male)	0.769 (0.504–1.172)	0.221			0.722 (0.478–1.090)	0.121		
**ECOG PS**		0.295				0.519		
0	Reference	–			Reference	–		
1	1.361 (0.912–2.032)	0.132			1.252 (0.846–1.852)	0.261		
2	1.399 (0.441–4.437)	0.568			1.214 (0.384–3.843)	0.741		
**CEA level** (Elevated vs. normal)	1.870 (1.219–2.866)	0.004			1.742 (1.147–2.646)	0.009		
**CA 19-9 level** (Elevated vs. normal)	1.658 (1.049–2.622)	0.031			1.942 (1.357–2.780)	0.041		
**Tumour location**		0.005				0.007		
Cardia of the stomach	Reference	–			Reference	–		
Body of the stomach	0.756 (0.449–1.272)	0.291			0.838 (0.509–1.381)	0.489		
Antrum of the stomach	0.504 (0.331–0.776)	0.001			0.532 (0.353–0.801)	0.003		
**Tumour size** (>4 cm vs. ≤4 cm)	2.142 (1.475–3.109)	<0.001			2.194 (1.529–3.149)	<0.001		
**Tumour grade**		0.060				0.079		
Grade 1	Reference	–			Reference	–		
Grade 2	4.529 (1.068–19.215)	0.040			5.176 (1.228–21.812)	0.025		
Grade 3	5.344 (1.312–21.764)	0.019			5.433 (1.336–22.103)	0.018		
Grade 4	7.291 (1.645–32.324)	0.009			7.176 (1.619–31.819)	0.009		
**Lauren type** (Diffuse and mixed vs. intestinal)	1.700(1.159–2.494)	0.007			1.579 (1.092–2.281)	0.015		
**Depth of invasion**		<0.001		0.018		<0.001		0.049
T1	Reference	–	Reference	–	Reference	–	Reference	–
T2	3.442 (0.822–14.401)	0.091	2.256 (0.529–9.623)	0.272	2.129 (0.616–7.354)	0.232	1.437 (0.409–5.052)	0.572
T3	7.039 (1.962–25.248)	0.003	3.228 (0.872–11.947)	0.079	4.639 (1.633–13.177)	0.004	2.415 (0.828–7.042)	0.106
T4a	12.661 (3.995–40.125)	<0.001	4.688 (1.415–15.533)	0.011	8.172 (3.312–20.163)	<0.001	3.198 (1.233–8.296)	0.017
T4b	24.761 (7.151–85.744)	<0.001	7.282 (1.967–26.956)	0.003	14.135 (5.120–39.025)	<0.001	4.175 (1.408–12.383)	0.010
**Lymph node metastasis**		<0.001		<0.001				<0.001
N0	Reference	–	Reference	–	Reference	–	Reference	–
N1	2.372 (1.266–4.445)	0.007	1.548 (0.817–2.932)	0.180	2.078 (1.148–3.762)	0.016	1.345 (0.732–2.470)	0.340
N2	4.353 (2.402–7.889)	<0.001	2.533 (1.364–4.702)	0.003	3.745 (2.136–6.567)	<0.001	2.257 (1.251–4.071)	0.007
N3a	8.563 (4.818–15.218)	<0.001	3.874 (2.111–7.107)	<0.001	8.264 (4.816–14.180)	<0.001	3.875 (2.174–6.909)	<0.001
N3b	12.189 (6.566–22.629)	<0.001	5.973 (3.057–11.671)	<0.001	10.779 (5.935–19.577)	<0.001	4.753 (2.477–9.120)	<0.001
**Distant metastasis** (M1 vs. M0)	3.540 (1.887–6.643)	<0.001	2.611 (1.350–5.051)	<0.001	3.450 (1.840–6.466)	<0.001	2.518 (1.306–4.854)	0.006
**PS**_**GC**_	4.263 (3.054–5.952)	<0.001	2.209 (1.539–3.170)	<0.001	4.281 (3.084–5.943)	<0.001	2.372 (1.646–3.420)	<0.001

Association of all variables with prognosis is analysed using a two-sided Cox proportional hazard regression analysis.

*PS*_*GC*_ pathomics signature of gastric cancer, *CEA* carcinoembryonic antigen, *CA* carbohydrate antigen, *HR* hazard ratio, *CI* confidence interval, *ECOG PS* Eastern Cooperative Oncology Group performance status.

In the training cohort, the pathomics nomogram yielded a concordance index (C-index) of 0.809 (95% CI: 0.741–0.878) for OS and 0.792 (95% CI: 0.718–0.866) for DFS. In addition, the time-dependent receiver operating characteristic (ROC) curve of the pathomics nomogram at 5 years produced an area under the receiver operating characteristic curve (AUROC) of 0.901 (95% CI: 0.863–0.939) for OS and 0.891 (95% CI: 0.850–0.932) for DFS (Supplementary Fig. 15a, b). Furthermore, the calibration curves showed good agreement between the nomogram-predicted survival and actual survival (Supplementary Fig. 16a, b). The good discrimination with a C-index of 0.784 (95% CI: 0.706–0.862) for OS and 0.794 (95% CI: 0.709–0.873) for DFS was externally validated in the validation cohort. The AUROCs for OS and DFS were 0.887 (95% CI: 0.842–0.931) and 0.888 (95% CI: 0.844–0.933), respectively (Supplementary Fig. 15c, d). The favourable agreement between the nomogram-predicted survival and actual survival of the calibration curves was also confirmed in the validation cohort (Supplementary Fig. 16c, d). Finally, the decision curve analysis indicated that using the pathomics nomograms to predict OS and DFS provided more net benefits than using the treat all scheme or treat none scheme in both the training and validation cohorts (Supplementary Fig. 17), indicating that the pathomics nomograms were clinically applicable.

### Incremental value of the PS_GC_ added to the TNM stage model

Two TNM stage models for OS and DFS were built based on multivariate Cox regression analyses without the PS_GC_ to elucidate the incremental value of the PS_GC_ added to clinicopathological variables for predicting the prognosis (Supplementary Table 5). In the training cohort, the C-index of the PS_GC_ for the prediction of OS and DFS was 0.727 (95% CI: 0.641–0.813) and 0.712 (95% CI: 0.622–0.802), respectively, and the TNM stage models showed a C-index of 0.782 (95% CI: 0.709–0.855) for OS and 0.770 (95% CI: 0.694–0.846) for DFS. Compared with the TNM stage models, the pathomics nomograms, which were based on the combination of PS_GC_ and the TNM staging system, displayed a significantly improved C-index of 0.809 (95% CI: 0.741–0.878; *P* = 0.002) for OS and 0.792 (95% CI: 0.718–0.866; *P* = 0.022), respectively (Supplementary Table 6). Similarly, the AUROCs of the PS_GC_ for OS and DFS were 0.798 (95% CI: 0.744–0.852) and 0.794 (95% CI: 0.739–0.848), respectively, and the TNM stage models yielded an AUROC of 0.868 (95% CI: 0.825–0.910) for OS and 0.859 (95% CI: 0.814–0.904) for DFS. Compared with the TNM stage models, the pathomics nomograms exhibited a significantly higher AUROC of 0.901 (95% CI: 0.863–0.939; *P* = 0.004) for OS and 0.891 (95% CI: 0.850–0.932; *P* = 0.005) for DFS (Supplementary Fig. 15a, b). The decision curve analysis indicated that compared with the TNM stage models, the pathomics nomograms showed greater net benefits across most of the range of reasonable threshold probabilities (Supplementary Fig. 17a, b). Moreover, the pathomics nomograms showed a net reclassification improvement (NRI) of 0.177 (95% CI: 0.021–0.319; *P* = 0.026) for OS and 0.218 (95% CI: 0.048–0.344; *P* = 0.012) for DFS compared to the TNM stage models (Supplementary Table 7). The abovementioned results were well validated in the validation cohort. In the validation cohort, the PS_GC_ demonstrated a C-index of 0.725 (95% CI: 0.627–0.823) for OS and 0.738 (95% CI: 0.642–0.834) for DFS, respectively, and the TNM stage models presented a C-index of 0.742 (95% CI: 0.656–0.828) for OS and 0.748 (95% CI: 0.660–0.836) for DFS. Compared with the TNM stage models, a significantly increased C-index of 0.784 (95% CI: 0.706–0.862; *P* < 0.001) for OS and 0.794 (95% CI: 0.709–0.873; *P* < 0.001) for DFS was observed in the pathomics nomograms (Supplementary Table 6). Meanwhile, the AUROCs of the PS_GC_ for OS and DFS were 0.774 (95% CI: 0.710–0.837) and 0.775 (95% CI: 0.711–0.839), respectively, and the TNM stage models exhibited an AUROC of 0.848 (95% CI: 0.797–0.900) for OS and 0.846 (95% CI: 0.794–0.898) for DFS. Compared with the TNM stage models, a significantly enhanced AUROC of 0.887 (95% CI: 0.842–0.931; *P* = 0.003) for OS and 0.888 (95% CI: 0.844–0.933; *P* = 0.003) for DFS was also confirmed in the pathomics nomograms (Supplementary Fig. 15c, d). In addition, higher net benefits across most of the range of reasonable threshold probabilities in the pathomics nomograms compared to the TNM stage models were detected (Supplementary Fig. 17c, d). Finally, an NRI of 0.318 (95% CI: 0.147–0.497; *P* = 0.010) for OS and 0.380 (95% CI: 0.141–0.556; *P* = 0.028) for DFS in the pathomics nomograms compared to the TNM stage models was found in the validation cohort (Supplementary Table 7). Herein, the PS_GC_ could provide additional prognostic value to the TNM staging system for GC.

### Predictive value of the PS_GC_ for adjuvant chemotherapy response

**Fig. 4:**
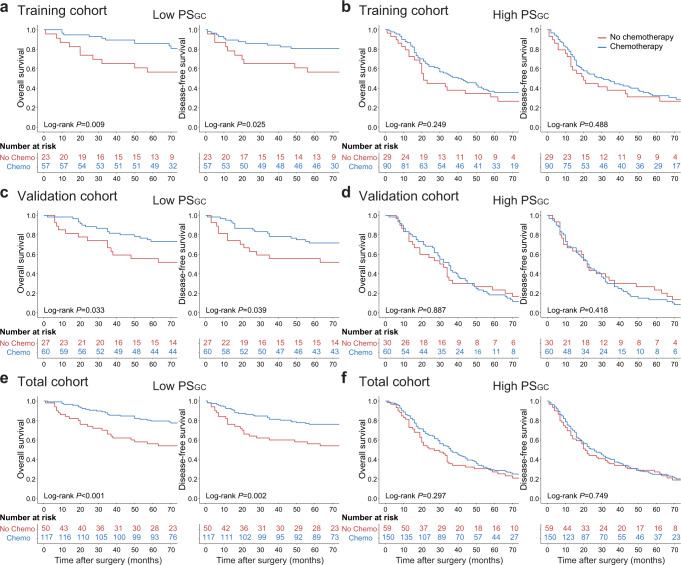
Association between the PS_GC_ and survival benefits from adjuvant chemotherapy in stage II and stage III GC. **a** Survival benefits from adjuvant chemotherapy for the low-PS_GC_ patients in the training cohort. **b** Survival benefits from adjuvant chemotherapy for the high-PS_GC_ patients in the training cohort. **c** Survival benefits from adjuvant chemotherapy for the low-PS_GC_ patients in the validation cohort. **d** Survival benefits from adjuvant chemotherapy for the high-PS_GC_ patients in the validation cohort. **e** Survival benefits from adjuvant chemotherapy for the low-PS_GC_ patients in the total cohort. **f** Survival benefits from adjuvant chemotherapy for the high-PS_GC_ patients in the total cohort. The comparisons of OS and DFS between the two groups are performed using a two-sided log-rank test. PS_GC_ pathomics signature of gastric cancer, GC gastric cancer, Chemo chemotherapy. Source data are provided as a Source data file.

To assess the predictive value of the PS_GC_ for adjuvant chemotherapy response, we evaluated the association between the PS_GC_ and survival among GC patients with stage II and stage III disease who either received or did not receive postoperative adjuvant chemotherapy. Patient information after stratification according to adjuvant chemotherapy status is listed in Supplementary Table 8. For the low-PS_GC_ patients, adjuvant chemotherapy was significantly associated with improved OS and DFS in the training, validation and total cohorts; however, the improved prognosis was not observed in high-PS_GC_ patients (Fig. 4). Similar results were obtained from the subgroup analyses of patients with stage II and III tumours (Supplementary Figs. 18, 19). No difference in the performance status of patients with a high PS_GC_ to tolerate the full course of chemotherapy was observed (Supplementary Table 9). Subsequently, a test of the interaction between the PS_GC_ and adjuvant chemotherapy indicated that patients with a low PS_GC_ had superior adjuvant chemotherapy benefits compared to patients with a high PS_GC_, with the *P* for interaction <0.05 for OS and DFS (Table 3, Supplementary Table 10). Taken together, these results indicated that the PS_GC_ could identify stage II and III GC patients who might obtain survival benefits from adjuvant chemotherapy.

**Table 3:** Association of the PS_GC_ with overall survival and disease-free survival in stage II and III patients receiving adjuvant chemotherapy

PS_GC_ level	Adjuvant chemotherapy		Overall survival			Disease-free survival		
	No chemo	Chemo	HR (95% CI)	*P*	*P*_interaction_	HR (95% CI)	*P*	*P*_interaction_
**Training cohort (*n*** = **199)**								
High PS_GC_ (Chemo vs. No chemo)	29	90	0.748 (0.454–1.233)	0.255	0.001	0.840 (0.513–1.377)	0.490	<0.001
Low PS_GC_ (Chemo vs. No chemo)	23	57	0.366 (0.136–0.786)	0.013		0.388 (0.165–0.914)	0.030	
**Validation cohort (*n*** = **177)**								
High PS_GC_ (Chemo vs. No chemo)	30	60	1.028 (0.639–1.657)	0.908	<0.001	1.203 (0.753–1.924)	0.439	<0.001
Low PS_GC_ (Chemo vs. No chemo)	27	60	0.459 (0.221–0.955)	0.037		0.475 (0.231–0.979)	0.044	
**Total cohort (*n*** = **376)**								
High PS_GC_ (Chemo vs. No chemo)	59	150	0.834 (0.591–1.177)	0.302	<0.001	0.947 (0.675–1.329)	0.751	<0.001
Low PS_GC_ (Chemo vs. No chemo)	50	117	0.392 (0.224–0.687)	0.001		0.434 (0.250–0.755)	0.003	

Association of all variables with prognosis is analysed using a two-sided Cox proportional hazard regression analysis.

*PS*_*GC*_ pathomics signature of gastric cancer, *HR* hazard ratio, *CI* confidence interval, *Chemo* chemotherapy.

## Discussion

Accurate prediction of prognosis and adjuvant chemotherapy benefits is integral to the risk stratification and management of GC patients in the clinic. In this study, we constructed the PS_GC_ to predict the prognosis of patients with GC and found that the PS_GC_ successfully stratified patients into high- and low-PS_GC_ groups with significant differences in terms of OS and DFS. Furthermore, by combining the PS_GC_ and TNM staging systems, we developed and validated two pathomics nomograms with significantly improved prognostic predictions compared with the TNM staging system alone. These results indicate that the PS_GC_ might provide complementary information about the prognosis of GC.

Adjuvant chemotherapy is a standard treatment for nonmetastatic advanced GC^3,19^. However, the variations in survival outcomes even in patients with the same TNM stage who receive the same regimens indicate that a considerable number of patients do not benefit from adjuvant chemotherapy. Individualized biomarkers that can distinguish patients who are likely to benefit from adjuvant chemotherapy could improve tailored therapy^20^. Our results revealed that patients with a low PS_GC_ were predicted to benefit from adjuvant chemotherapy, but in patients with a high PS_GC_, limited benefits were observed. Patient performance status is an important factor that might affect tolerance to adjuvant chemotherapy. In this study, we did not observe a difference in the performance status of patients with a high PS_GC_ to tolerate the full course of chemotherapy, indicating that the predictive value of the PS_GC_ for adjuvant chemotherapy benefits might be also applicable to patients with a poor physical condition. Although patients receiving neoadjuvant therapy were excluded, most patients included in this study were still diagnosed with locally advanced GC, which did not imply that patients with a potentially lower risk were included. To our knowledge, this is the first study to demonstrate the utility of fully quantitative imaging features extracted from H&E-stained slides to predict prognosis and benefits from adjuvant chemotherapy in GC.

Two factors were critical for the construction of the PS_GC_. The first factor is the use of a convenient image-processing approach to extract quantitative pathomics features. To date, a consensus about the extraction of pathomics features has not been reached^12,13^. CellProfiler is a free, open-source software that automatically measures phenotypes from biological images and has been used in digital pathology analysis with satisfactory performance^21,22^. Therefore, CellProfiler is an easy-to-use and reproducible platform that allows clinicians to extract quantitative pathomics features. The second factor is a practical machine learning method for the selection of prognostic features^23^. For this purpose, the LASSO-Cox regression model was employed because of its ability to deal with high-dimensional data^16,17^.

Despite its limited performance, the TNM staging system remains the cornerstone for predicting the prognosis of patients with GC. To date, some investigators have explored potential biomarkers that might provide additional prognostic information in GC. Based on the gene expression data, several molecular classifications have been proposed. For example, according to The Cancer Genome Atlas project, GC was divided into four subtypes based on molecular classification, including tumours positive for Epstein-Barr virus, microsatellite unstable tumours, genomically stable tumours and tumours with chromosomal instability, which might aid in patient stratification and tailoring therapy^5,7^. However, the cost and complexity of gene expression data analyses prevent their clinical application, especially in developing countries. In addition, a radiomics analysis of radiological images has also shown a favourable ability for predicting the prognosis of GC^24,25^. Other studies have also revealed that the stromal immune cells, such as tumour-associated macrophages, cytotoxic T cells and neutrophils, are indicators of the GC prognosis^26,27^. Prospective studies and further evaluations are needed to better clarify their impacts on the prognosis of GC. In addition to the pathological diagnosis, the evaluation of H&E-stained sections has provided limited information on patient prognosis and chemotherapy response. Currently, the literature regarding the prognostic information of pathomics analysis in GC has not yet been reported. In this study, we discovered that the PS_GC_ could also contribute to the prediction of prognosis and identify patients who are more likely to benefit from adjuvant chemotherapy in GC. Because the PS_GC_ was derived from the routinely used H&E-stained sections in the clinic, the PS_GC_ might be conveniently applied in clinical practice without additional financial burden and might favour the development of tailored therapy for GC. We expect that these biomarkers, including molecular subtypes, radiomics, stromal immune cells, and pathomics, will be utilized together to improve the prediction of prognosis and chemotherapy response of GC in the future.

In Western countries, patients with locally advanced GC are recommended to receive neoadjuvant chemotherapy because of the prolonged survival^28,29^; however, radical gastrectomy followed by adjuvant chemotherapy remained the standard of care for these patients in Eastern Asia^2,30^, and patients with locally advanced GC are treated with this therapeutic strategy in our medical centre^31^. Patients receiving neoadjuvant chemotherapy were excluded from our study, as neoadjuvant chemotherapy would result in morphological changes in the H&E-stained sections, including tumour regression and fibrosis; thus, the prediction models developed in this study might be inappropriate to be extended to patients with GC who receive neoadjuvant chemotherapy. However, our results revealed that the pathomics analysis reflected tumour heterogeneity, which was a potential indicator of the prognosis and chemotherapy response of patients with GC. Thus, the pathomics analysis might also be suitable for evaluating the response and outcomes of neoadjuvant chemotherapy. Further investigations are required in this specific setting.

Pathomics is a novel method that has been utilized to explore tumour heterogeneity since different degrees of disease progression, clinical outcomes, and treatment responses correspond to a range of histologic features in different tumour cells^11^. Traditional pathological examination is performed by experienced pathologists at multiple magnifications to evaluate the characteristics of tumour cells; however, pathologists do not and cannot routinely characterize more detailed information for every slide. Thus, pathomics can serve as a useful method to complement traditional pathological evaluation^32^. Our results revealed significant differences in the Lauren type and tumour grade subgroups, indicating that the local image features would change according to the Lauren type and tumour grade, and the Lauren type and tumour grade might drive the PS_GC_. Moreover, a similar distribution of PS_GC_ was found between the tumour size subgroups, which implied no bias in the selection of regions of interest due to tumour size.

The PS_GC_ was found to be a potential predictor of prognosis and adjuvant chemotherapy benefits for GC patients. However, it remains unclear whether the predictive value of the PS_GC_ is determined by tumour intrinsic factors or tumour microenvironment effects. Currently, the integrative analysis of pathomics features and genomics data provides a feasible way to explore the underlying mechanisms of PS_GC_ with prognosis and adjuvant chemotherapy benefits^33,34^. Thus, further investigations should focus on the relationship between pathomics features and genomics data.

The tumour grade is a common term used to diagnose GC in the clinic, which evaluates the progression of tumour cells. Our data showed that the PS_GC_ substantially outperformed the tumour grade in predicting the OS and DFS (Supplementary Table 11 and Supplementary Fig. 20). The prognostic performance of the TNM staging system was not increased when the tumour grade was added; conversely, significantly improved prognostic performance was detected when the PS_GC_ was added to the TNM staging system (Supplementary Table 12 and Supplementary Fig. 21). Thus, the PS_GC_ performed better when it was included in the pathomics nomogram than when it was replaced by the tumour grade.

The improvement in AUROC ranged from 3.2% to 4.2% when the PS_GC_ was added to the TNM staging system, and the corresponding improvement in the C-index ranged from 2.2% to 4.6%, which might seem small. In this study, the pathomics nomograms were developed based on the depth of invasion, lymph node metastasis, distant metastasis and PS_GC_. In terms of the individual variables, the prognostic value of the PS_GC_ was comparable to that of the lymph node metastasis (Supplementary Tables 13 and 14). However, in the pathomics nomograms, lymph node metastasis had the most important contribution to predict the prognosis, followed by the PS_GC_. Based on these results, lymph node metastasis exerted more powerful effects on the AUROCs and C-indexes of the pathomics nomograms than the PS_GC_, although the individual prognostic performance of the two variables was comparable, which explained the small incremental value of adding PS_GC_ to the TNM staging system. Several prognostic biomarkers with statistically different but numerically small incremental values have also been reported previously^35–37^. Although the incremental value of adding PS_GC_ to the TNM staging system for predicting the prognosis was small, it did provide additional prognostic information. Meanwhile, the ability of the PS_GC_ to predict response to adjuvant chemotherapy was valuable, which might avoid the toxic effects of chemotherapy in those patients least likely to benefit. Thus, from the perspective of clinicians, the PS_GC_ was clinically relevant and worth further investigation.

The AUROCs of the pathomics nomograms were acquired from the multivariate analysis of potential prognostic factors. Currently, a unanimous consensus has not been reached about the calculation of sample size in the multivariate analysis for developing a prediction model. According to the TRIPOD Statement, at least 10 events per variable are needed^38^. Thus, the minimum sample size of patients with recurrence is 100 according to the 10 events per variable criteria to assess the difference in AUROCs between the pathomics nomograms and TNM stage models. In the training cohort, 122 patients suffered from recurrence. Thus, our sample size in the training cohort was adequate to conduct the multivariate analysis. For the sample size in the validation cohort, Lei et al.^39^ suggested that the ratio between the training and validation cohorts was 7:3. In our study, the validation cohort contained 216 patients, which was also sufficient.

Considering the survival differences between the training and validation cohorts (OS: 58.7% vs. 47.7%; DFS: 55.3% vs. 45.4%), we speculated that it might be due to the socioeconomic differences despite the similar clinicopathological characteristics between the two cohorts. The training cohort and validation cohort came from Guangdong and Fujian in China, respectively. Guangdong is the most economically advanced province in China, most patients in the training cohort live in urban areas, and the local medical insurance covers more examinations and therapies; conversely, the economic level of Fujian is moderate in China, and considerable numbers of patients in the validation cohorts reside in rural areas, and the local medical insurance covers the costs of fewer examinations and therapies^40^. Therefore, despite the rigorous follow-up after surgery, early detection and treatment of recurrent diseases are limited for patients in the validation cohort, thus resulting in differences in survival.

According to the Global Burden of Diseases, Injuries, and Risk Factors Study (GBD) 2017 Stomach Cancer Collaborators, the estimated 5-year OS of GC is approximately 20%, with the exceptions of 65% in Japan and 71·5% in South Korea, where population screening has led to the effective diagnosis of tumours at early stages^1^. In this study, all included GC patients who had received radical gastrectomy; however, the data sources of the GBD 2017 were derived from patients diagnosed with GC, regardless of resectable or unresectable diseases. Thus, the survival outcomes of the included patients are high by global standards. In addition, the 5-year OS of GC patients receiving radical gastrectomy generally ranges from 45% to 60% in China, indicating that the survival outcomes of patients with GC included in this study are similar to those in other parts of China^30^.

Our results revealed that elevated CEA and CA 19-9 levels were significantly associated with a worse prognosis. In clinical practice, CEA and CA 19-9 levels are the most common tumour markers measured before surgery and during follow-up for GC. CEA and CA 19-9 levels have been used as diagnostic markers and are apt to rise 2–3 months before metastatic lesions become detectable by imaging modalities^2^. Several studies have also reported that elevated preoperative CEA and CA 19-9 levels are associated with a worse prognosis for patients with resectable GC^41,42^. Currently, the intrinsic mechanisms of elevated CEA and CA 19-9 levels for worse survival are still unclear. One possible explanation might be that CEA and CA 19-9, which are a ligand of E-selectin and an intercellular adhesion molecule, respectively, play critical roles in the intercellular adhesion of tumour cells to vascular endothelial cells and contribute to tumour invasion and metastasis^43,44^. Thus, the prognostic values of CEA and CA 19-9 levels for GC need to be further investigated.

In general, there are two main types of artificial intelligence-based computational approaches for pathomics analysis: deep neural network-based approaches and handcrafted feature-based approaches^12^. The method used in this study is a handcrafted feature-based approach that was developed based on the close collaboration between pathologists and oncological surgeons, and thus could be complex and time-consuming^45^. Deep neural network-based approaches are developed through unsupervised feature learning, which depends on the existence of learning sets and annotated exemplars from the categories of interest, and the network design usually focuses on fine-tuning the algorithm to maximize accuracy while minimizing processing time^46^. In addition, deep neural network-based approaches trained on a particular disease subtype could be applied to other subtypes as well^12^. However, in terms of interpretability, because of being more interpretable than deep neural network-based approaches, handcrafted feature-based approaches might be more likely to be used for high-level decision-making, such as that regarding oncological prognosis or prediction of benefit from therapy; in contrast, deep neural network-based approaches might be more appropriate in situations where the need to “explain the decision” is reduced; such situations could include low-level tasks such as object detection or segmentation^12,47,48^. Considering the application scene of this study and that oncologists and pathologists are the primary end users, we select the handcrafted feature-based approaches.

There are some limitations in our study. First, given the retrospective design, our study was not free from inherent biases. Second, all enroled participants came from two medical centres in China. Thus, further validation in prospective randomized trials incorporating diverse populations is warranted to test the clinical utility of the PS_GC_ for individualized decision-making.

In conclusion, our study constructed the PS_GC_ and found that the PS_GC_ was significantly associated with the prognosis of patients with GC. By integrating the PS_GC_ with the TNM staging system, we developed and validated two pathomics nomograms, which improved the prediction of the GC prognosis compared to the TNM staging system alone. Moreover, the PS_GC_ could distinguish patients with stage II and III diseases who were likely to derive benefits from adjuvant chemotherapy.

## Methods

This study was approved by the Institutional Review Boards of Nanfang Hospital of Southern Medical University and the Fujian Cancer Hospital of Fujian Medical University. Written informed consent was obtained from all patients before surgery, which contained a statement on the formalin-fixed, paraffin-embedded samples and clinicopathological data for scientific research. All procedures involving human participants were in accordance with the Declaration of Helsinki.

### Participants

A retrospective cohort study was conducted based on consecutive patients who underwent radical gastrectomy for GC from two medical centres. A training cohort including 264 consecutive patients from March 2012 to December 2013 at Nanfang Hospital of Southern Medical University was built. The inclusion criteria were as follows: (i) histologically diagnosed GC and treated with curative surgery, (ii) at least 15 lymph nodes were harvested, (iii) no history of other malignancies, and (iv) complete clinicopathological and follow-up information were available. Patients receiving neoadjuvant chemotherapy, radiotherapy or chemoradiotherapy were excluded because neoadjuvant anticancer therapy not only results in microscopic morphological changes in the H&E-stained sections, including tumour regression and fibrosis but also affects the prognosis of patients with GC. A total of 216 consecutive patients were included from Fujian Provincial Cancer Hospital of Fujian Medical University between August 2010 and September 2012 using the same inclusion and exclusion criteria.

Patient baseline information, including age, sex, Eastern Cooperative Oncology Group performance status (ECOG PS), CEA level, CA 19-9 level, tumour location, tumour size, tumour grade, Lauren type, depth of invasion, lymph node metastasis, distant metastasis, TNM stage, postoperative adjuvant chemotherapy and follow-up data (follow-up duration and survival status), was collected. The TNM stage was reclassified according to the eighth version of the *AJCC Cancer Staging Manual* of the American Joint Committee on Cancer. Patients were followed up once every 3 months in the first 2 years after surgery, every 6 months in the next 3 years, and annually thereafter. The follow-up duration was measured from the time of surgery to the last follow-up date, and the survival status at the last follow-up was recorded. OS was defined as the interval between surgery and death or the last date of follow-up. DFS was defined as the time from surgery to recurrence at any site or all-cause death, whichever came first.

### Sample preparation and region of interest selection

The H&E-stained slides of all included patients were prepared using formalin-fixed paraffin-embedded samples. Then, sections most representative of the depth of invasion in each case were selected by the director of the Department of Pathology, Fujian Cancer Hospital (G.C.), who had 25 years of experience in the pathological diagnosis of GC. Subsequently, all selected slides were scanned by using the Aperio ScanScope Scanner system (Leica Biosystems) with the ×20 objective, and images were digitized as svs. format files, which were managed with the Aperio ImageScope software (version 12.4.6). Under the quality control of the director of the Department of Pathology, Fujian Cancer Hospital (G.C.), the tumour areas in each section were determined. Ten nonoverlapping representative tiles of each case containing the greatest number of tumour cells with a field of view of 1000 × 1000 pixels (one pixel is equal to 0.504 μm) were selected by a pathologist and then confirmed by the other pathologist (L.L. and J.L.), who had 9 and 13 years of experience in the pathological diagnosis of GC, respectively, to reduce the computational time. The regions of tissue folds were excluded and the selected tiles were saved as.tif format files. If the abovementioned two pathologists differed in their opinions, they consulted with the third pathologist (G.C.) to make a decision.

### Extraction of pathomics features from images

The quantitative pathomics features of the selected tiles were extracted by using CellProfiler (version 4.0.7), an open-source image analysis software developed by the Broad Institute (Cambridge, MA)^21,22^. The H&E-stained images were split into haematoxylin-stained and eosin-stained greyscale images using the “UnmixColors” module^49^. The H&E-stained images were also converted to greyscale images using the “ColorToGray” module based on the “Combine” method for further analysis. First, the features that indicated the image quality of the greyscale H&E, haematoxylin and eosin images were assessed by using the “MeasureImageQuality” and “MeasureImageIntensity” modules with three types of features, including blurred features, intensity features and threshold features^50–53^. The threshold features were extracted by automatically calculating the threshold for each image to identify the tissue foreground from the unstained background with the Otsu algorithm^54^. Subsequently, the colocalization and correlation between intensities in each haematoxylin-stained image and eosin-stained image were calculated on a pixel-by-pixel basis across an entire image by using the *“*MeasureColocalization” module^55^. In addition, the granularity features of each image were assessed using the “MeasureGranularity” module, which outputted spectra of size measurements of the textures in the image, with a granular spectrum range of 16^56,57^. Further description of the pipeline for feature extraction is described in the Supplementary Methods. A summary of the pathomics features is presented in Supplementary Table 15.

### Construction of the PS_GC_

The LASSO-Cox regression model uses an L1 penalty to shrink the coefficients of each feature to zero, and this model has been broadly applied in the regression analysis of high-dimensional data for survival analysis^16,17^. The penalty parameter *λ*, also called the tuning constant, controls the strength of the penalty. If *λ* is reduced and the penalty is relaxed, then more predictors can enter the model. In this study, 10-fold cross-validation with minimum criteria was used to determine the optimum value of *λ* by measuring partial likelihood deviance in the training cohort. The PS_GC_ was constructed via a linear combination of the selected features, and the PS_GC_ for the validation cohort was directly calculated from the formula obtained in the training cohort.

### Association of the PS_GC_ with prognosis

Based on the individual PS_GC_, an optimum cutoff value was identified via the maximally selected rank statistics to classify patients into high- and low-PS_GC_ groups in the training cohort, and then the same cutoff value was applied to the validation cohort^58^. Potential associations of the PS_GC_ with OS and DFS were first assessed in the training cohort and then validated in the validation cohort. The differences in the survival curves of the high- and low-PS_GC_ groups were evaluated.

### Development and validation of the pathomics nomogram for prognosis

In the training cohort, the PS_GC_ and clinicopathological characteristics were incorporated into the univariate Cox regression analyses for OS and DFS. Variables with *P* < 0.05 were selected for the multivariate Cox regression analyses. The backwards stepwise regression was utilized to detect the independent predictors. Two pathomics nomograms including the independent predictors were developed to predict OS and DFS, respectively.

To quantify the discrimination performance, the C-index and 5-year AUROC were calculated^59^. To compare the agreement between predicted survival probabilities and the actual probabilities, calibration curves were generated^60^. To evaluate the clinical usefulness of the pathomics nomograms, decision curve analysis was used to assess the net benefits of the prediction model at different threshold probabilities^61,62^. The context for decision curve analysis is a situation where individuals’ risks for an undesirable outcome will be evaluated, and individuals with sufficiently high risk will be recommended for some intervention or treatment. The pathomics nomograms were then applied in the validation cohort to validate the discrimination, calibration, and clinical usefulness^38^.

### Incremental value of the PS_GC_ for prognosis prediction

Two clinicopathological models incorporating independent clinicopathological risk factors for the prediction of OS and DFS were developed in the training cohort, respectively, and then applied to the validation cohort to determine the incremental value of the PS_GC_ for the individualized prediction of the prognosis when added to the clinicopathological risk factors. The incremental value of the PS_GC_ to the clinicopathological models was assessed with respect to the C-index, time-independent AUROC, NRI, and decision curve analysis^38^. The C-indexes and time-independent AUROCs at 5 years between the two models were compared by using the *z*-score test and DeLong test, respectively^63,64^. The NRI of the pathomics nomogram to the clinicopathological model was assessed by using the *Z* test^65^.

### Statistical analysis

Continuous variables were compared using the independent samples, unpaired *t*-test if they were normally distributed or using the Mann–Whitney *U* test if they were nonnormally distributed. When comparing a categorical variable, if at least one expected cell count was <5 in the 2 × *C* contingency table, Fisher’s exact test was used; otherwise, the *χ*^2^ test was performed. Survival curves were generated using the Kaplan–Meier method and compared using the log-rank test. Cox regression analysis was used for univariate and multivariate analyses, and the hazard ratio (HR) with 95% CI was calculated. The PH assumption was checked for the Cox regression models by constructing test statistics based on asymptotically mean-zero processes^66^. If the global *P* < 0.05, the PH assumption was violated; otherwise, the assumption was valid^66^. The relative importance of each variable in the multivariable model to predict the prognosis was assessed by using the *χ*^2^ statistic minus the corresponding degree of freedom^67^. Interactions between the PS_GC_ and adjuvant chemotherapy were detected by means of Cox regression analysis. Assessment of the effects of PS_GC_ on adjuvant chemotherapy benefits in stage II and III GC patients was prespecified. All statistical analyses were performed using SPSS (version 19.0) and R (version 4.0.5) software. The LASSO-Cox regression method was performed using the “*glmnet*” package. The PH assumption was checked using the “*CoxPhLb*” package. The development, validation and performance assessment of the prognostic nomograms were conducted using the “*rms*” package. Comparisons of C-indexes between different models were performed using the “*compareC*” package. The time-dependent ROC curves were plotted using “*riskRegression*” package. Decision curve analysis was performed with the function of “*stdca.R*”. The “*survminer*” package was used for computing survival analyses. Tests were 2-sided, and *P* < 0.05 was considered to indicate statistical significance.

### Reporting summary

Further information on research design is available in the Nature Portfolio Reporting Summary linked to this article.

## Supplementary information

Supplementary Information

Reporting Summary

## Data Availability

The H&E images and clinical information analysed during the current study are not publicly available for patient privacy purposes. Data access can be obtained through a reasonable request to J.Y. (yanjunfudan@163.com). Access to the data will be restricted to non-commercial research which removes patient-sensitive information. All requests will be answered within 10 working days. The source data underlying Figs. 2–4 and Supplementary Figs. 1–21 is provided as a Source data file. Source data are provided with this paper.

## Source data

Source Data

## Code availability

The codes used for the slide image process and feature extraction are publicly available at https://github.com/CellProfiler/CellProfiler^22^. The codes used to develop and evaluate the prediction model are publicly available at https://github.com/Dexin-Chen/Pathomics-analysis and are also placed on the Zenodo platform^68^.
