# Supplementary material for: Prognostic and predictive value of a pathomics signature in gastric cancer
Source: Nat Commun. 2022 Nov 12;13:6903. doi: 10.1038/s41467-022-34703-w (PMC9653436; doi:10.1038/s41467-022-34703-w)
Supplement: Supplementary file 1 — Supplementary Information [file 41467_2022_34703_MOESM1_ESM.pdf]

Supplementary Information

Prognostic and predictive value of a pathomics signature in gastric cancer

Authors: Dexin Chen, Meiting Fu, Liangjie Chi, Liyan Lin, Jiaxin Cheng, Weisong Xue, Chenyan Long, Wei Jiang, Xiaoyu Dong, Jian Sui, Dajia Lin, Jianping Lu, Shuangmu Zhuo, Side Liu, Guoxin Li, Gang Chen, and Jun Yan

Supplementary Information

Supplementary Methods

Supplementary Figures

Supplementary Note

Supplementary Table

Supplementary References

Supplementary Methods

1. Pipeline for extracting pathomics features using CellProfiler

CellProfiler is a free, open-source software for the quantitative analysis of biological images (https://cellprofiler.org/)[1, 2]. The haematoxylin and eosin (H&E)-stained images were split into haematoxylin-stained and eosin-stained greyscale images using the *"UnmixColors"* module[3]. The H&E-stained images were also converted to greyscale images using the *"ColorToGray"* module based on the *"Combine"* method for further analysis.

First, the features that indicated the image quality of the greyscale H&E, haematoxylin and eosin images were assessed by using the *"MeasureImageQuality"* module with three types of features, including blur features, intensity features and threshold features[4-7]. The intensity features were also measured by using the "*MeasureImageIntensity*" module.

(1) Blur features

FocusScore: A measure of the intensity variance across the image. This score is calculated using a normalized variance, which is the best-ranking algorithm for brightfield, phase contrast, and differential interference contrast images[7]. A higher focus score corresponds to a lower blur.

LocalFocusScore: A measure of the intensity variance between image subregions. A local version of the focus score subdivides the image into nonoverlapping tiles, computes the normalized variance for each, and takes the mean of these values as the final metric.

Correlation: A measure of the correlation of the image for a given spatial scale, and the spatial scale was set at 20 in this study. This is a measure of the image spatial intensity distribution computed across subregions of an image for the given spatial scale[6]. If an image is blurred, the correlation between neighbouring pixels becomes high, producing a high correlation value.

PowerLogLogSlope: The slope of the image log-log power spectrum. The power spectrum contains the frequency information of the image, and the slope gives a measure of image blur. A higher slope indicates a lower frequency component and hence more blur[5]. This metric is recommended for blur detection in most cases.

(2) Intensity metrics

MeanIntensity: Mean of all pixel intensity values.

MedianIntensity: Median of all pixel intensity values.

StdIntensity: Standard deviation of all pixel intensity values.

MADIntensity: Median absolute deviation (MAD) of all pixel intensity values. The MAD is defined as the median ($|x_i$ - median(x)$|$).

LowerQuartileIntensity: The intensity value of the pixel for which 25% of the pixels in the object have lower values.

UpperQuartileIntensity: The intensity value of the pixel for which 75% of the pixels in the object have lower values.

(3) Threshold metrics

Threshold: The automatically calculated threshold for each image to identify the tissue foreground from the unstained background with the Otsu algorithm[8].

Subsequently, the colocalization and correlation between intensities in each haematoxylin image and eosin image were calculated on a pixel-by-pixel basis across an entire image by using the *"MeasureColocalization"* module[9].

Correlation: The correlation between a pair of haematoxylin (H)- and eosin (E)-stained images was calculated as Pearson's correlation coefficient. The calculation formula is covariance (H, E) / [std(H) × std(E)].

Slope: The slope of the least-squares regression between a pair of H and E images. Calculated using the model $\alpha \times H + \beta = E$, where α is the slope.

Overlap coefficient: The overlap coefficient is a modification of Pearson's correlation coefficient in which the average intensity values of the pixels are not subtracted from the original intensity values. For a pair of H and E images, the formula used to calculate the overlap coefficient is sum ($H_i \times E_i$) / sqrt (sum ($H_i \times H_i$) × sum ($E_i \times E_i$)).

Manders coefficient: The Manders coefficient for a pair of H and E images is calculated as M1 = sum (H_i_coloc) / sum (H_i) and M2 = sum (E_i_coloc) / sum (E_i), where H_i_coloc = H_i when $E_i > 0$ or 0 otherwise, and E_i_coloc = E_i when $H_i > 0$ or 0 otherwise.

Manders coefficient (Costes' automated threshold): Costes' automated threshold estimates the maximum threshold of intensity for each image based on the *Correlation* value. The Manders coefficient is applied on thresholded images as H_i_coloc = H_i when $E_i > E_{thr}$ and E_i_coloc = E_i when $H_i > H_{thr}$, where E_{thr} and H_{thr} are thresholds calculated using Costes' automated threshold method[10].

Rank weighted colocalization coefficient: The rank weighted colocalization (RWC) coefficient for a pair of H and E images is measured as RWC1 = sum(H_i_coloc*W_i) / sum(H_i) and RWC2 = sum(E_i_coloc*W_i) / sum(E_i); in this formula, W_i is the weight defined as W_i = (H_{max} - D_i) / R_{max}, where R_{max} is the maximum ranks among H and E based on the maximum intensity, and D_i = abs(rank(H_i) - rank(E_i)) (absolute difference in ranks between H and R) and H_i_coloc = H_i when E_i > 0 or 0 otherwise and E_i_coloc = E_i when H_i >0 or 0 otherwise[11].

In addition, the granularity features of each image were calculated using the *"MeasureGranularity"* module, which outputted spectra of size measurements of the textures in the image, with a granular spectrum range of 16[12, 13].

Granularity: The module returns one measurement for each instance of the granularity spectrum set in the range of the granular spectrum.

Ultimately, a total of 90 features were extracted. The ultimate value of each quantitative feature was averaged from the 10 tiles for further statistical analyses.

Supplementary Figures

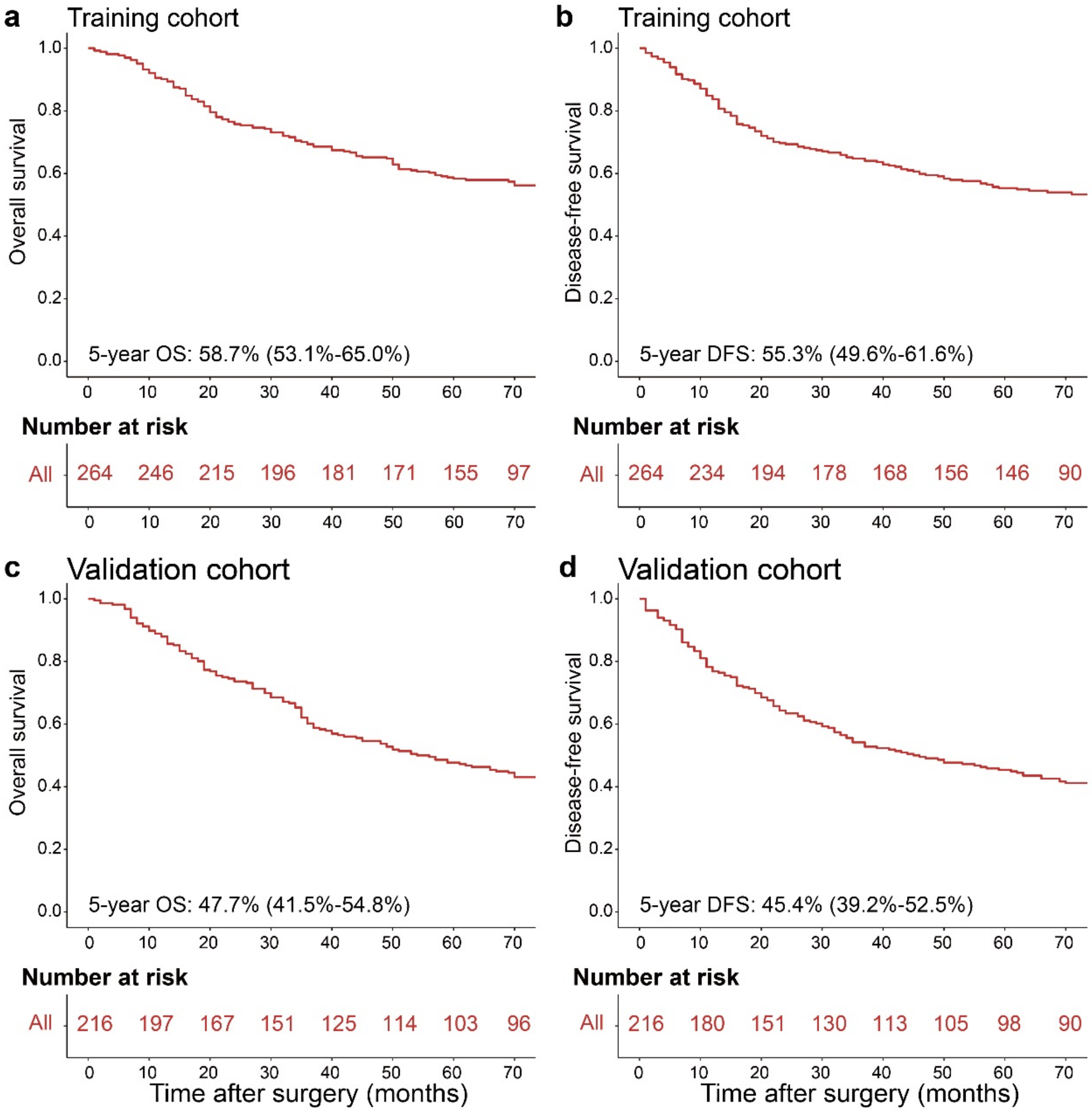

Supplementary Fig. 1. Kaplan-Meier survival analysis of the training and validation cohorts. **(a)** The OS curve in the training cohort. **(b)** The DFS curve in the training cohort. **(c)** The OS curve in the validation cohort. **(d)** The DFS curve in the validation cohort. OS, overall survival; DFS, disease-free survival. Source data are provided as a Source data file.

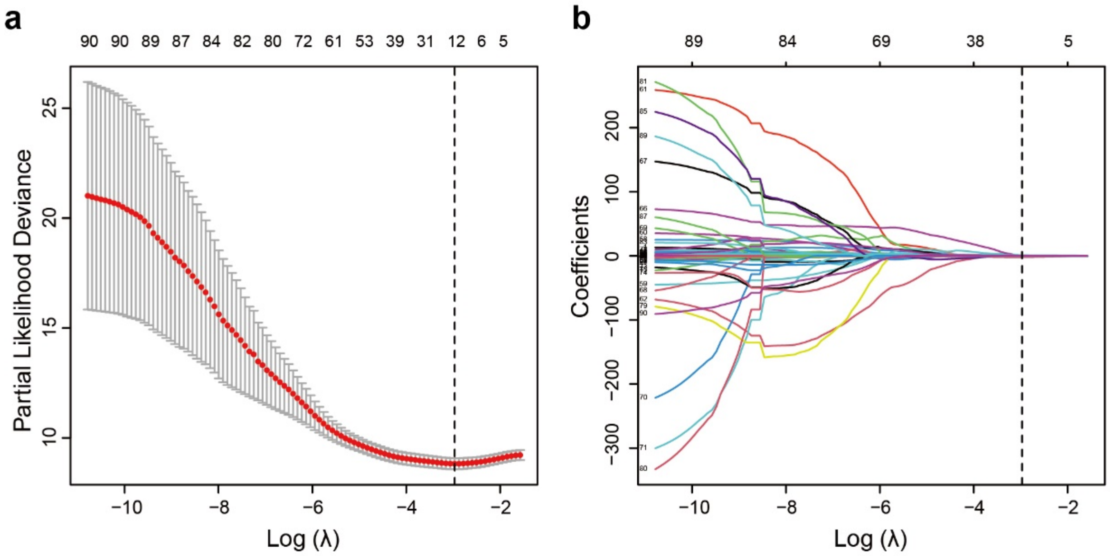

Supplementary Fig. 2. Feature selection using a LASSO-Cox regression model in training cohort. **(a)** Tuning parameter (λ) selection for the LASSO-Cox regression model via 10-fold cross-validation. The partial likelihood deviance is plotted versus the log (λ) value. Solid vertical lines represent the partial likelihood deviance ± SE. Dotted vertical line is shown at the optimal partial likelihood deviance values, which is calculated by using the minimum criteria. A λ value of 0.05129246, with a log (λ) value of -2.9702115162, is chosen by 10-fold cross-validation and the minimum criteria. In this cohort, n=264 patients. **(b)** Profiles of coefficients from the LASSO-Cox regression model of the extracted pathomics features. A dotted vertical line is shown at the value of log (λ) = -2.9702115162, at which the optimal λ value results in 12 nonzero coefficients. LASSO, least absolute shrinkage and selection operator; SE, standard error. Source data are provided as a Source data file.

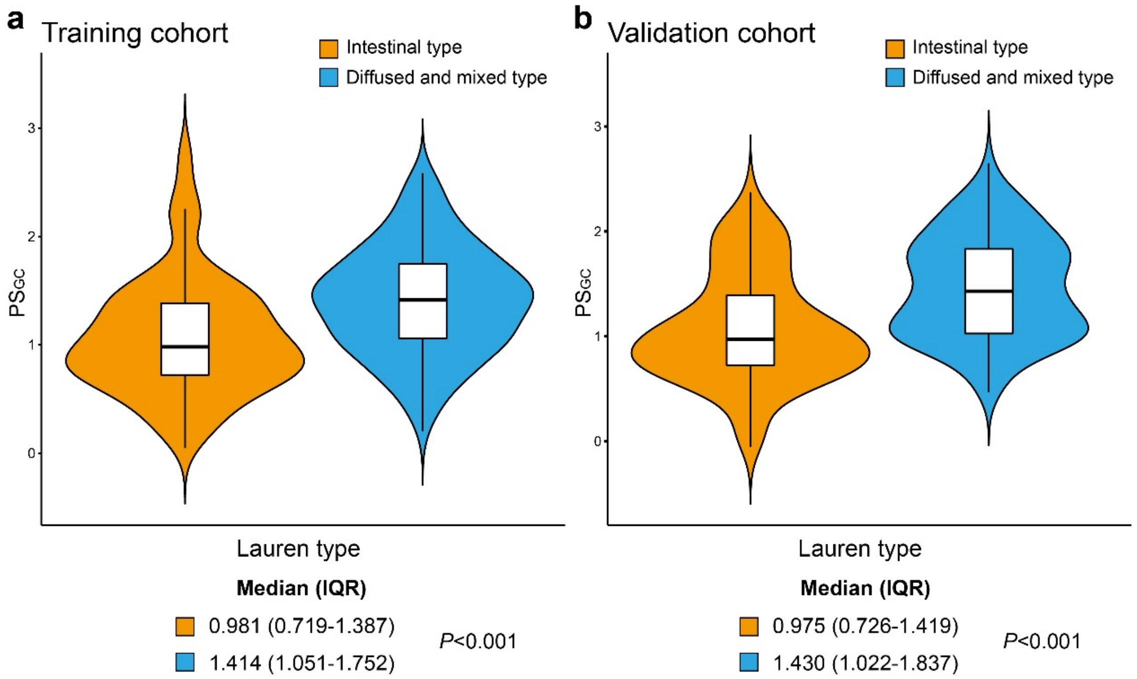

Supplementary Fig. 3. The distribution of PS_{GC} based on the Lauren type in the **(a)** training and **(b)** validation cohorts. In training cohort, n=120 and 144 for patients with intestinal type and diffused and mixed type, respectively. In validation cohort, n=92 and 124 for patients with intestinal type and diffused and mixed type, respectively. In violin plots, the centre lines are median, the upper and lower bounds of boxes indicate first and third quartiles, the whiskers mean the 1.5 times of interquartile range, and the upper and lower tails represent the maxima and minima, respectively. Data are analyzed by using a two-sided Mann-Whitney *U* test. PS_{GC}, pathomics signature of gastric cancer; IQR, interquartile range. Source data are provided as a Source data file.

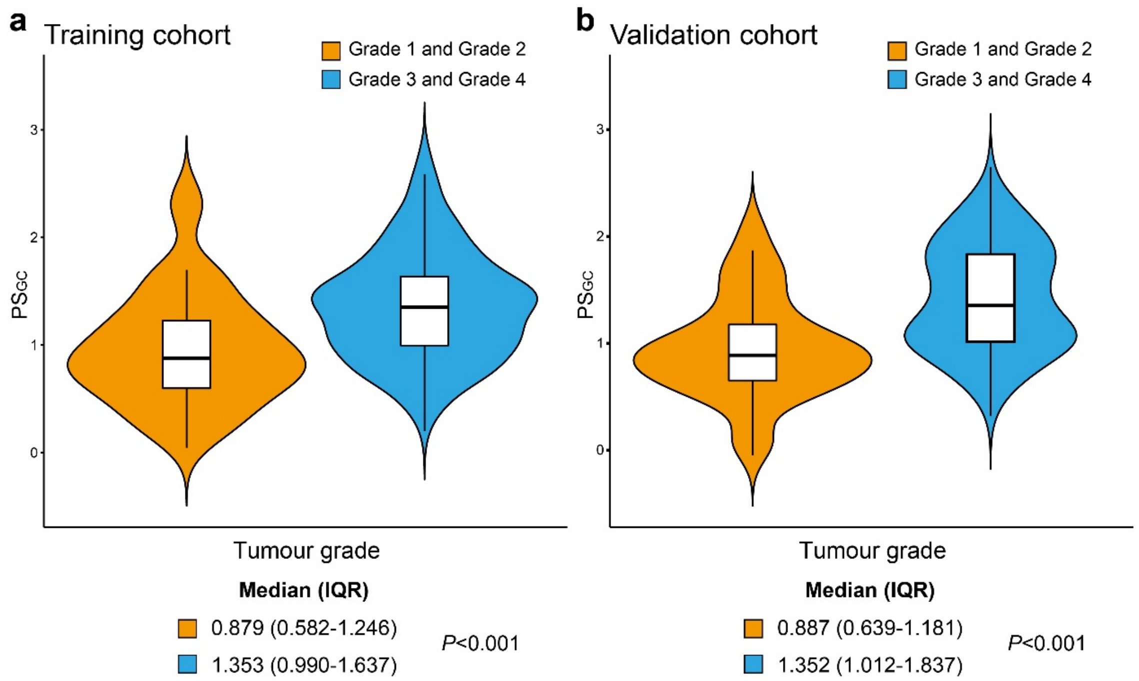

Supplementary Fig. 4. The distribution of PS_{GC} based on the tumour grade in the **(a)** training and **(b)** validation cohorts. In training cohort, n=72 and 192 for patients with Grade 1-2 and Grade 3-4, respectively. In validation cohort, n=56 and 160 for patients with Grade 1-2 and Grade 3-4, respectively. In violin plots, the centre lines are median, the upper and lower bounds of boxes indicate first and third quartiles, the whiskers mean the 1.5 times of interquartile range, and the upper and lower tails represent the maxima and minima, respectively. Data are analyzed by using a two-sided Mann-Whitney *U* test. PS_{GC}, pathomics signature of gastric cancer; IQR, interquartile range. Source data are provided as a Source data file.

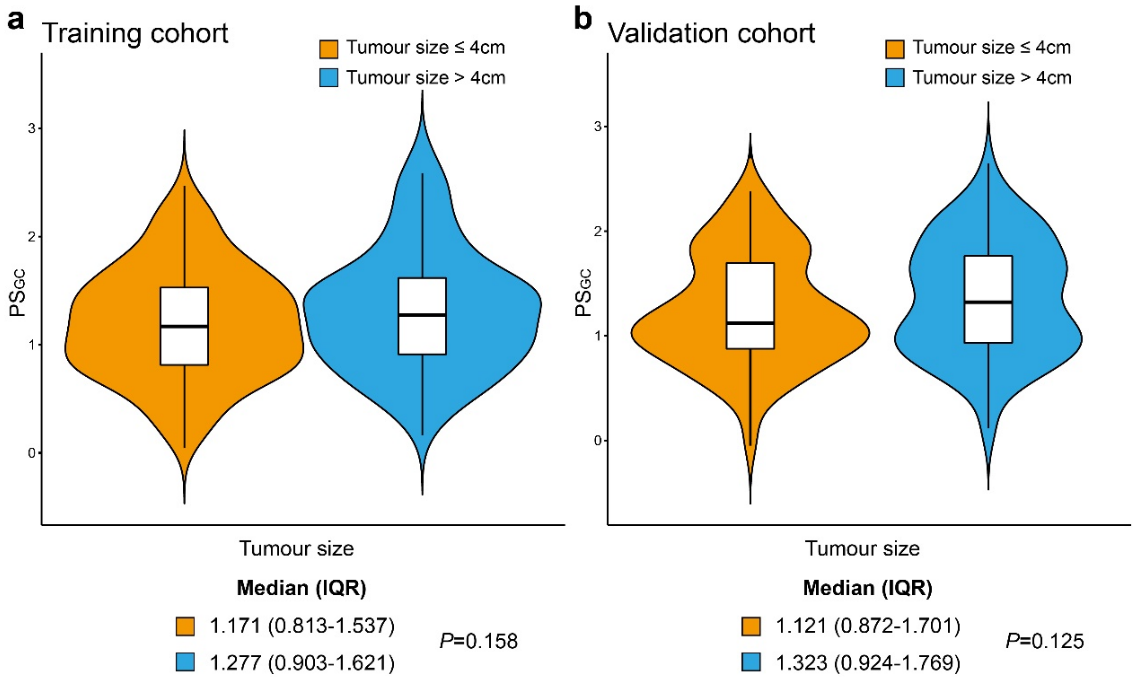

Supplementary Fig. 5. The distribution of PS_{GC} based on the tumour size in the **(a)** training and **(b)** validation cohorts. In training cohort, n=147 and 117 for patients with tumour size ≤4 cm and >4 cm, respectively. In validation cohort, n=110 and 106 for patients with tumour size ≤4 cm and >4 cm, respectively. In violin plots, the centre lines are median, the upper and lower bounds of boxes indicate first and third quartiles, the whiskers mean the 1.5 times of interquartile range, and the upper and lower tails represent the maxima and minima, respectively. Data are analyzed by using a two-sided Mann-Whitney *U* test. PS_{GC}, pathomics signature of gastric cancer; IQR, interquartile range. Source data are provided as a Source data file.

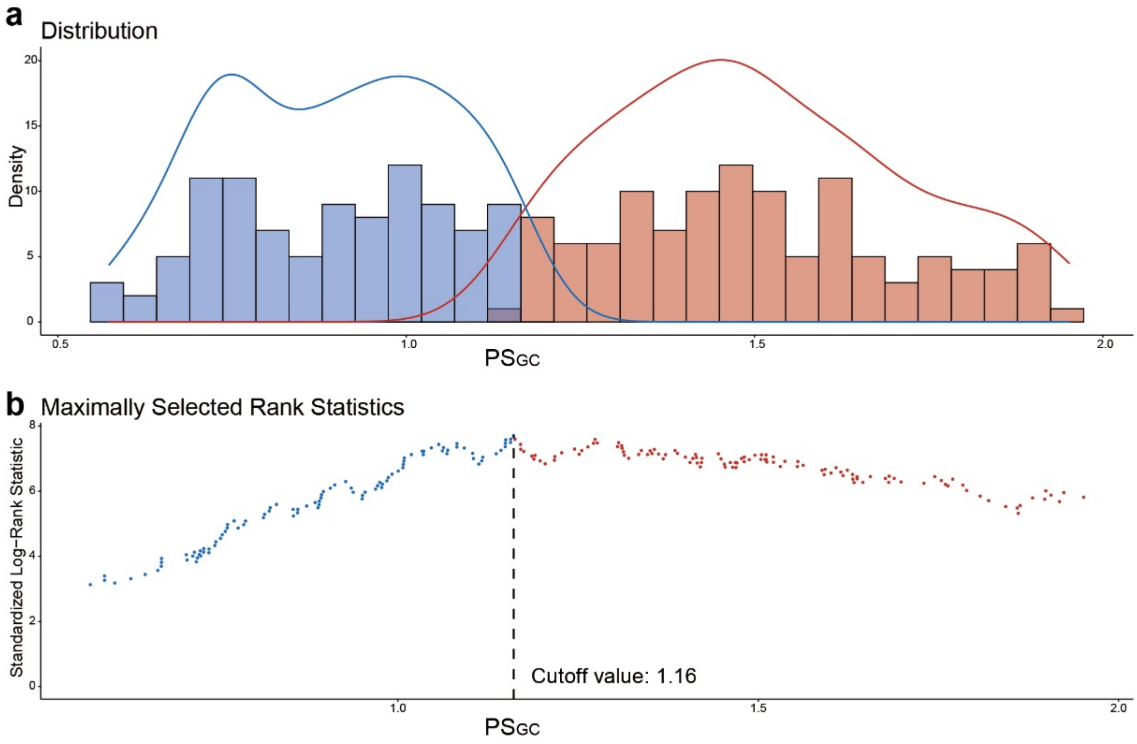

Supplementary Fig. 6. Selection of the optimum cutoff value for the PS_{GC}. **(a)** Histogram showing the density distribution for high- and low-PS_{GC} groups divided by the optimum cutoff value. **(b)** Scatter plot showing the standardized log-rank statistic value for each PS_{GC} cutoff value. In this cohort, n=141 and 123 for patients with high PS_{GC} and low PS_{GC}, respectively. The cutoff value is determined by using the two-sided maximally selected rank statistics. PS_{GC}, pathomics signature of gastric cancer. Source data are provided as a Source data file.

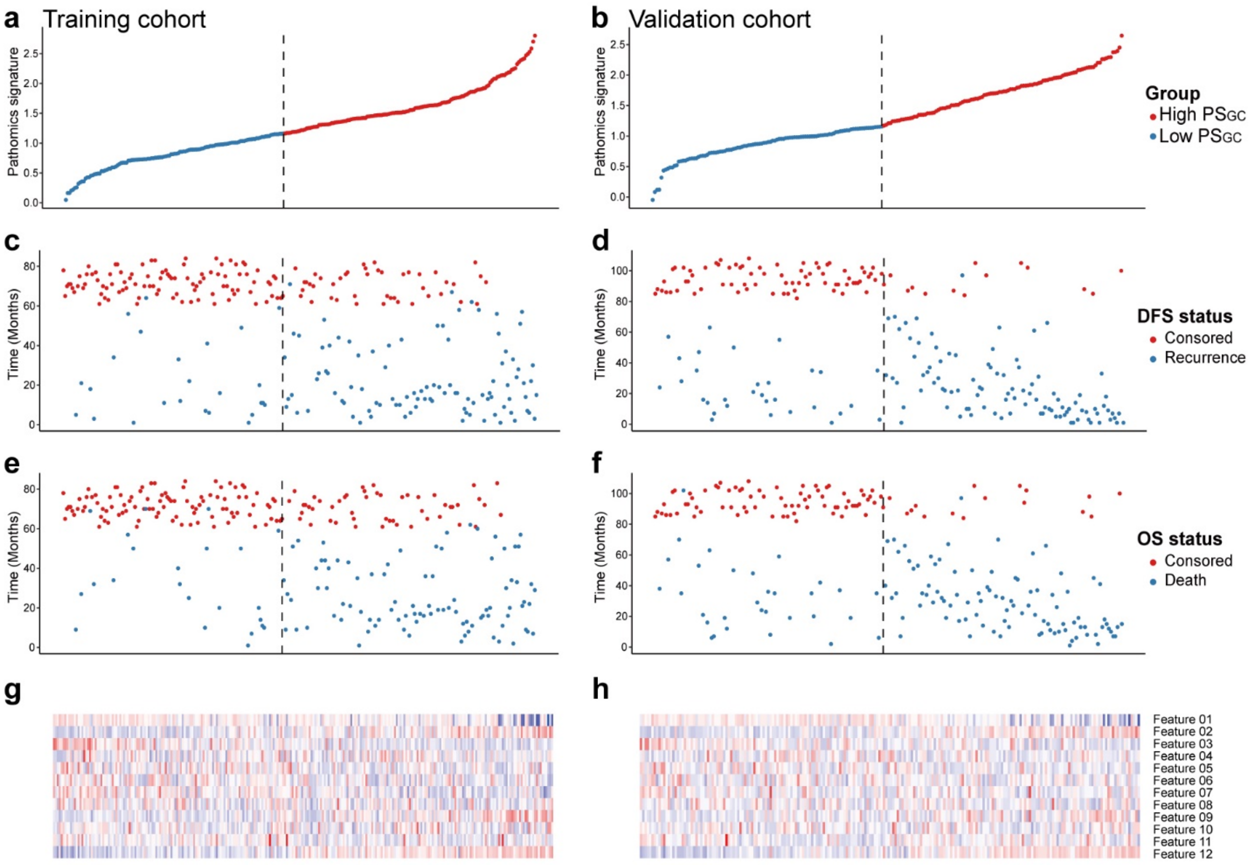

Supplementary Fig. 7. The distribution of the PS_{GC} with the corresponding survival status in the training and validation cohorts. **(a)** The distribution of the PS_{GC} in the training cohort. **(b)** The distribution of the PS_{GC} in the validation cohort. **(c)** The DFS status in the training cohort. **(d)** The DFS status in the validation cohort. **(e)** The OS status in the training cohort. **(f)** The OS status in the validation cohort. **(g)** Colour gram of the profiles of the selected pathomics features in the training cohort. **(h)** Colour gram of the profiles of the selected pathomics features in the validation cohort. The rows of the colour gram represent 12 pathomics features, and the columns represent patients. The vertical dashed line represents the cutoff value for the PS_{GC} that is used to divide the patients into high- and low-PS_{GC} groups. In training cohort, n=141 and 123 for patients with high PS_{GC} and low PS_{GC}, respectively. In validation cohort, n=110 and 106 for patients with high PS_{GC} and low PS_{GC}, respectively. PS_{GC}, pathomics signature of gastric cancer; DFS, disease-free survival; OS, overall survival. Source data are provided as a Source data file.

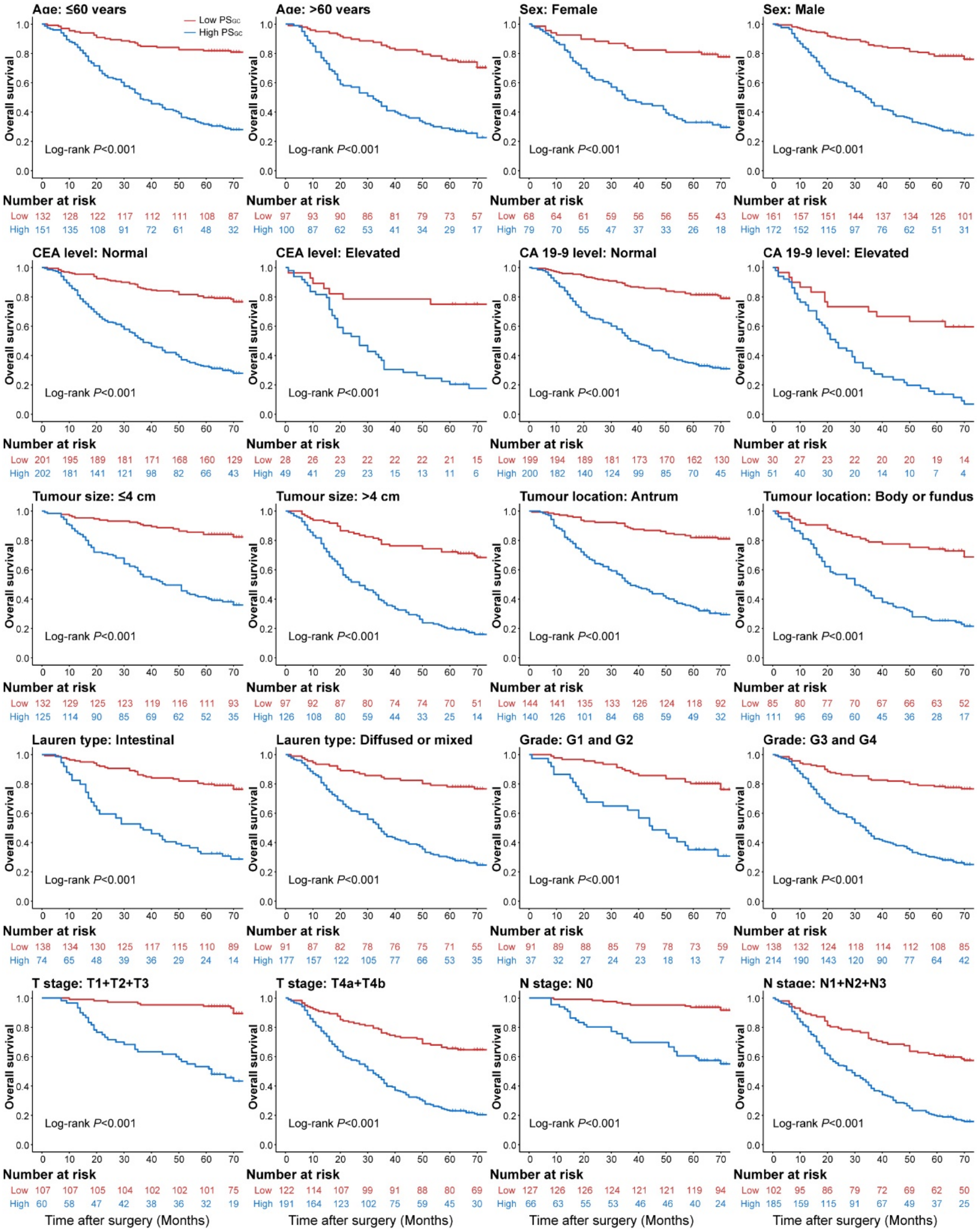

Supplementary Fig. 8. Kaplan-Meier survival analysis of the OS of all 480 patients according to the PS_{GC} level stratified by clinicopathological variables. The comparisons of OS between two groups are performed using a two-sided log-rank test. PS_{GC}, pathomics signature of gastric cancer; CEA, carcinoembryonic antigen; CA, cancer antigen; OS, overall survival. Source data are provided as a Source data file.

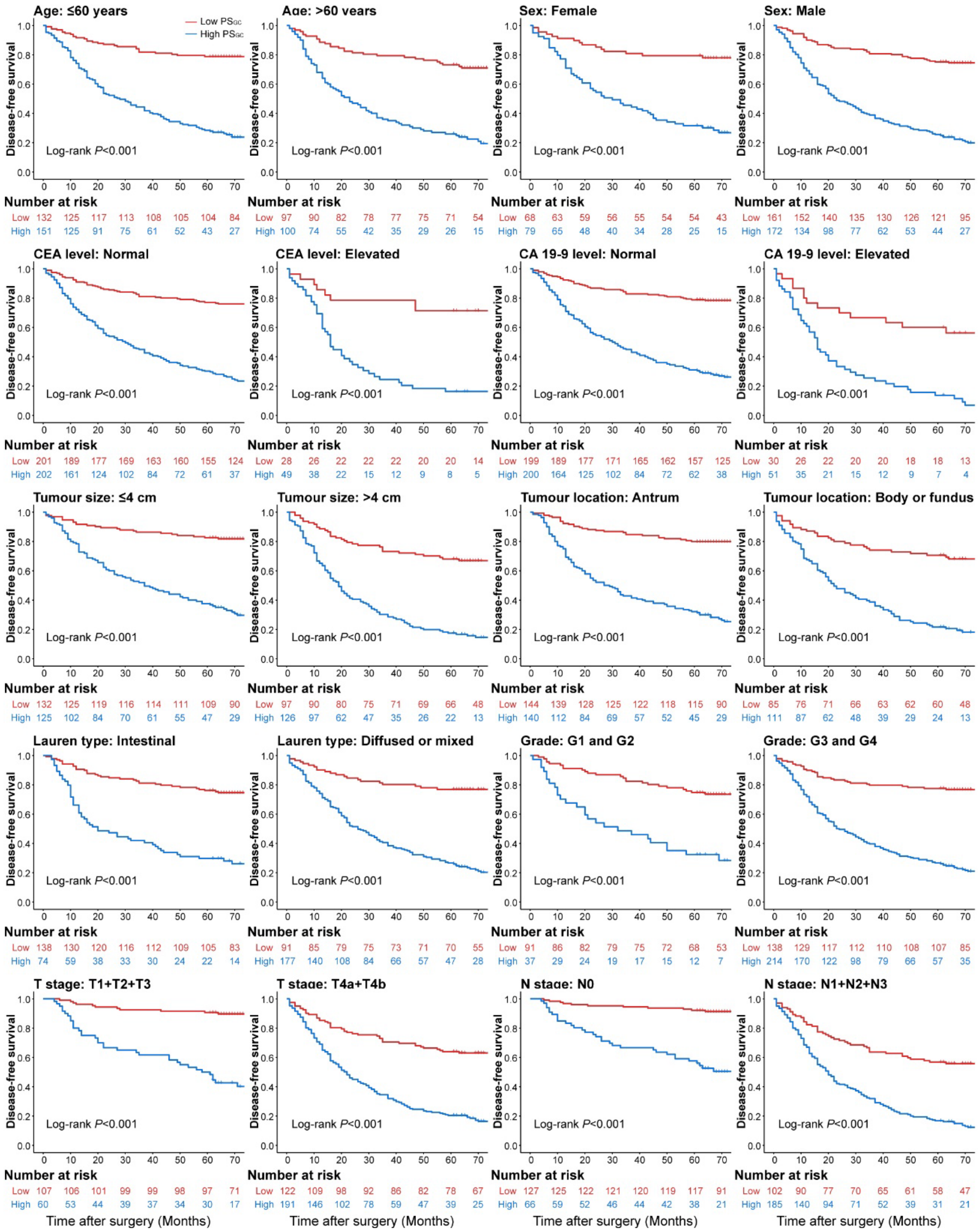

Supplementary Fig. 9. Kaplan-Meier survival analysis of the DFS of all 480 patients according to the PS_{GC} level stratified by clinicopathological variables. The comparisons of DFS between two groups are performed using a two-sided log-rank test. PS_{GC}, pathomics signature of gastric cancer; CEA, carcinoembryonic antigen; CA, cancer antigen; DFS, disease-free survival. Source data are provided as a Source data file.

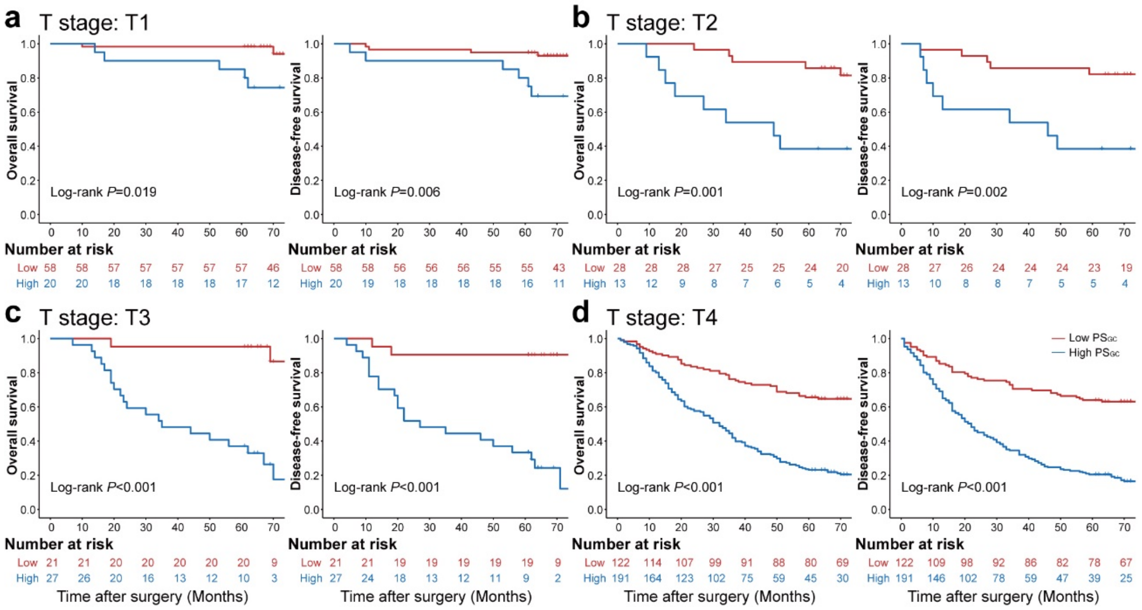

Supplementary Fig. 10. Kaplan-Meier survival analysis of OS and DFS according to the PS_{GC} level of patients stratified by the depth of invasion. **(a)** Kaplan-Meier survival analysis of OS and DFS according to the PS_{GC} level in the T1 subgroup. **(b)** Kaplan-Meier survival analysis of OS and DFS according to the PS_{GC} level in the T2 subgroup. **(c)** Kaplan-Meier survival analysis of OS and DFS according to the PS_{GC} level in the T3 subgroup. **(d)** Kaplan-Meier survival analysis of OS and DFS according to the PS_{GC} level in the T4 subgroup. The comparisons of OS and DFS between two groups are performed using a two-sided log-rank test. PS_{GC}, pathomics signature of gastric cancer; OS, overall survival; DFS, disease-free survival. Source data are provided as a Source data file.

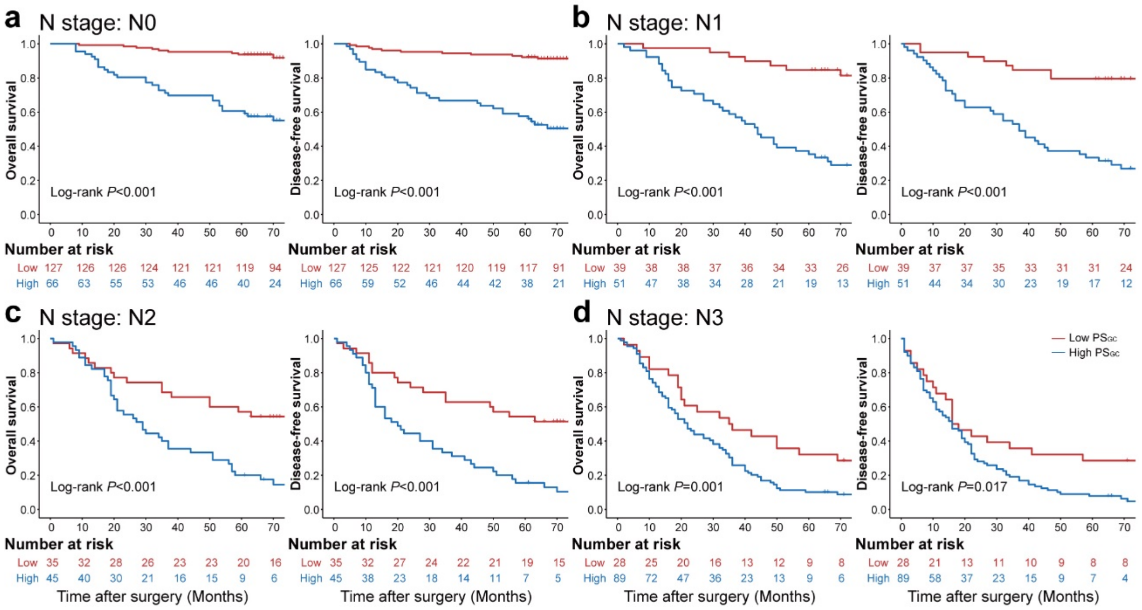

Supplementary Fig. 11. Kaplan-Meier survival analysis of OS and DFS according to the PS_{GC} level of patients stratified by lymph node metastasis. **(a)** Kaplan-Meier survival analysis of OS and DFS according to the PS_{GC} level in the N0 subgroup. **(b)** Kaplan-Meier survival analysis of OS and DFS according to the PS_{GC} level in the N1 subgroup. **(c)** Kaplan-Meier survival analysis of OS and DFS according to the PS_{GC} level in the N2 subgroup. **(d)** Kaplan-Meier survival analysis of OS and DFS according to the PS_{GC} level in the N3 subgroup. The comparisons of OS and DFS between two groups are performed using a two-sided log-rank test. PS_{GC}, pathomics signature of gastric cancer; OS, overall survival; DFS, disease-free survival. Source data are provided as a Source data file.

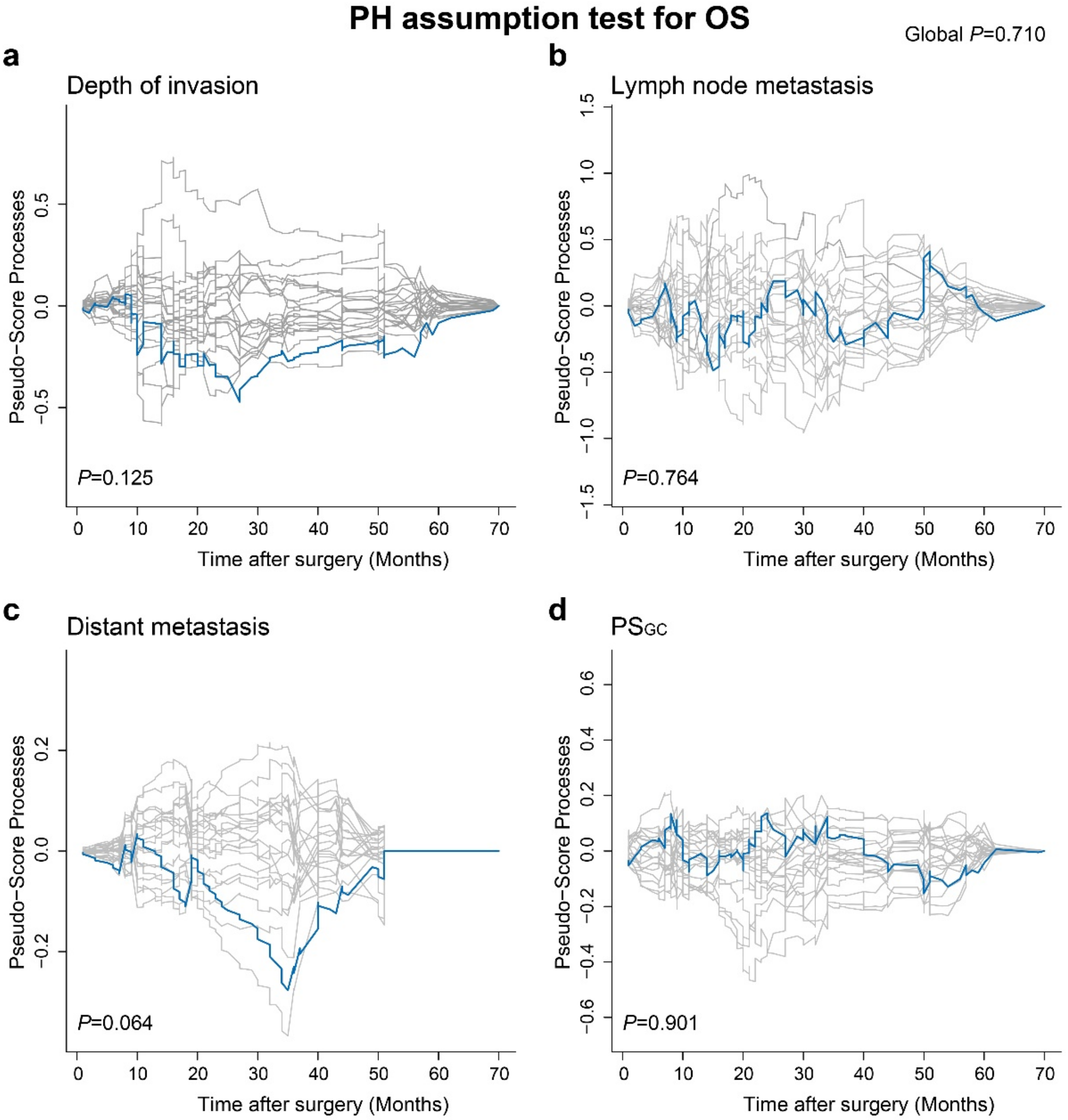

Supplementary Fig. 12. The PH assumption test of the multivariate Cox regression model for OS. **(a)** The PH assumption test of the depth of invasion for OS. **(b)** The PH assumption test of lymph node metastasis for OS. **(c)** The PH assumption test of distant metastasis for OS. **(d)** The PH assumption test of PS_{GC} for OS. In this cohort, n=264 patients. The PH assumption is checked using a two-sided asymptotical mean-zero test. PH, proportional hazards; PS_{GC}, pathomics signature of gastric cancer; OS, overall survival. Source data are provided as a Source data file.

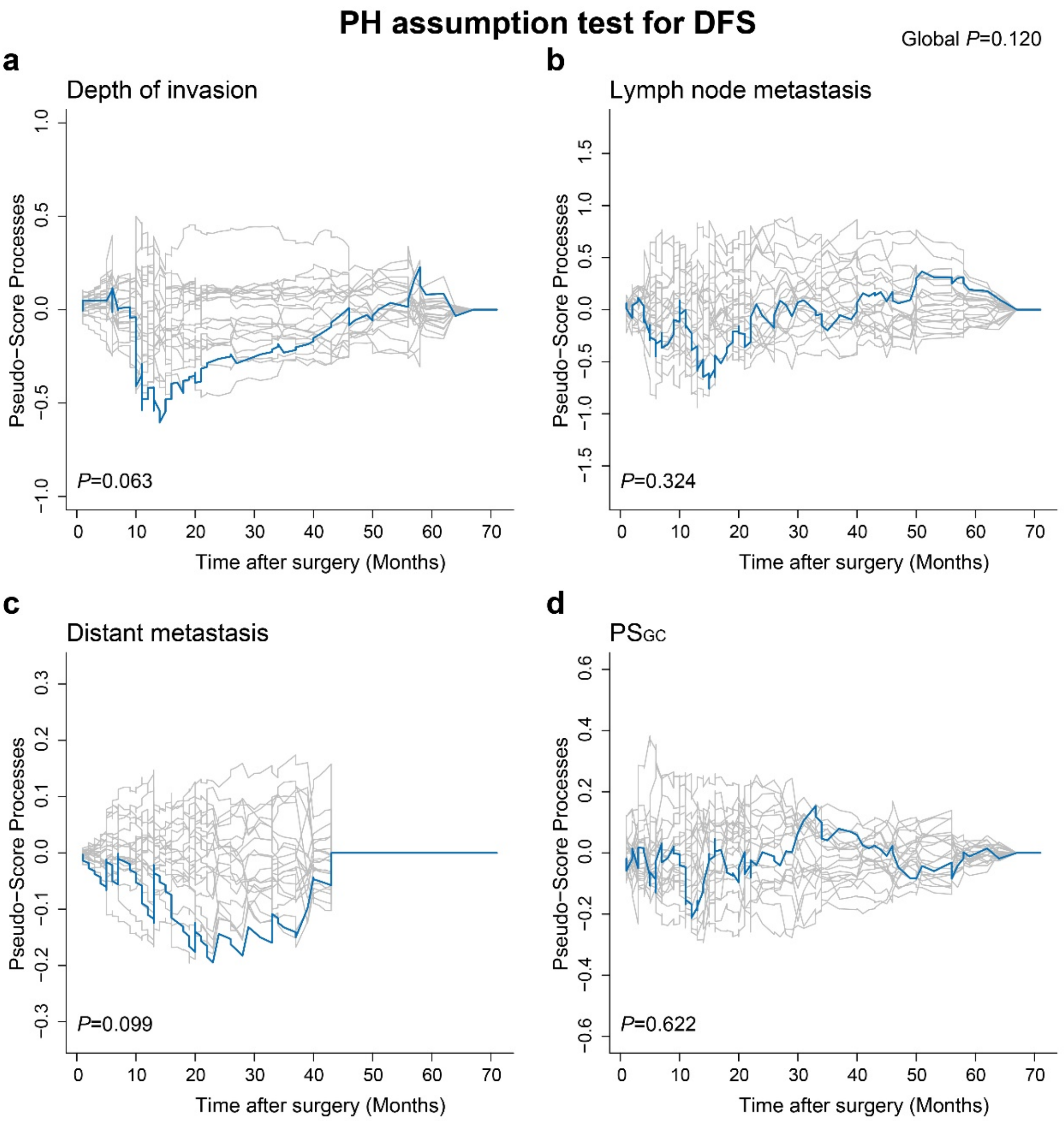

Supplementary Fig. 13. The PH assumption test of the multivariate Cox regression model for DFS. **(a)** The PH assumption test of the depth of invasion for DFS. **(b)** The PH assumption test of lymph node metastasis for DFS. **(c)** The PH assumption test of distant metastasis for DFS. **(d)** The PH assumption test of PS_{GC} for DFS. In this cohort, n=264 patients. The PH assumption is checked using a two-sided asymptotical mean-zero test. PH, proportional hazards; PS_{GC}, pathomics signature of gastric cancer; DFS, disease-free survival. Source data are provided as a Source data file.

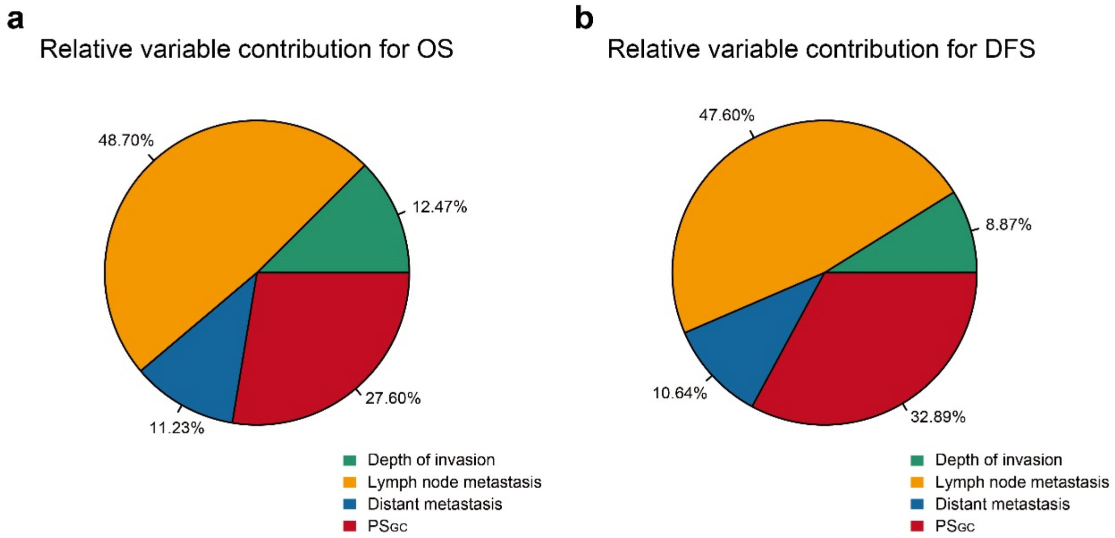

Supplementary Fig. 14. Relative importance of the variables in the pathomics nomogram. **(a)** Relative importance of each variable for OS in the pathomics nomogram. **(b)** Relative importance of each variable for DFS in the pathomics nomogram. PS_{GC}, pathomics signature of gastric cancer; OS, overall survival; DFS, disease-free survival. Source data are provided as a Source data file.

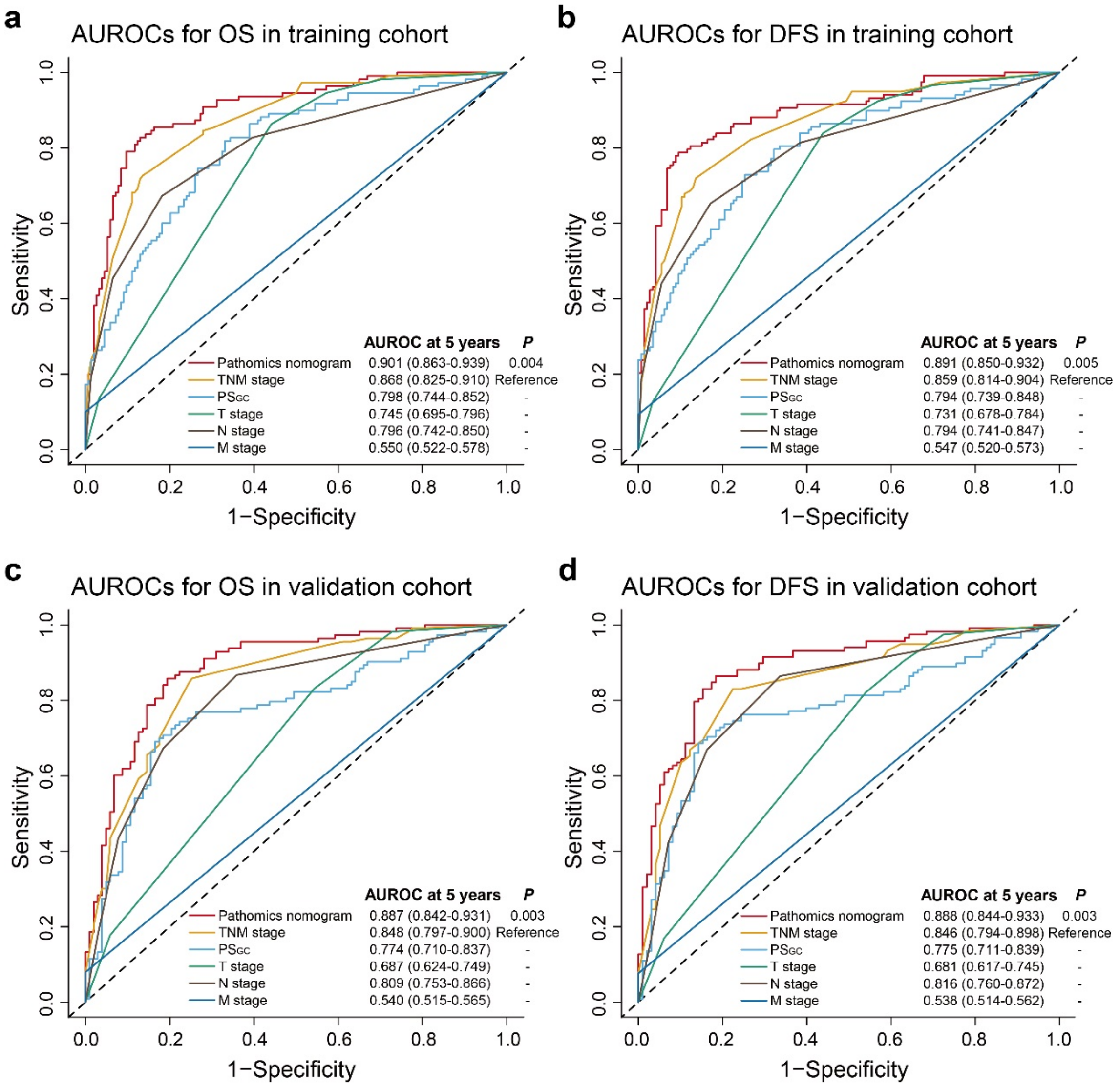

Supplementary Fig. 15. Time-independent ROC curves and AUROCs. **(a)** The time-independent ROC curves for OS with the AUROCs of different models in the training cohort. **(b)** The time-independent ROC curves for DFS with the AUROCs of different models in the training cohort. **(c)** The time-independent ROC curves for OS with the AUROCs of different models in the validation cohort. **(d)** The time-independent ROC curves for OS with the AUROCs of different models in the validation cohort. In training cohort, n=264 patients. In validation cohort, n=216 patients. The comparisons of AUROCs between two models are performed using a two-sided Delong test. ROC, receiver operating characteristic; AUROC, area under the receiver operating characteristic curve; OS, overall survival; DFS, disease-free survival; PS_{GC}, pathomics signature of gastric cancer; TNM, tumour-node-metastasis. Source data are provided as a Source data file.

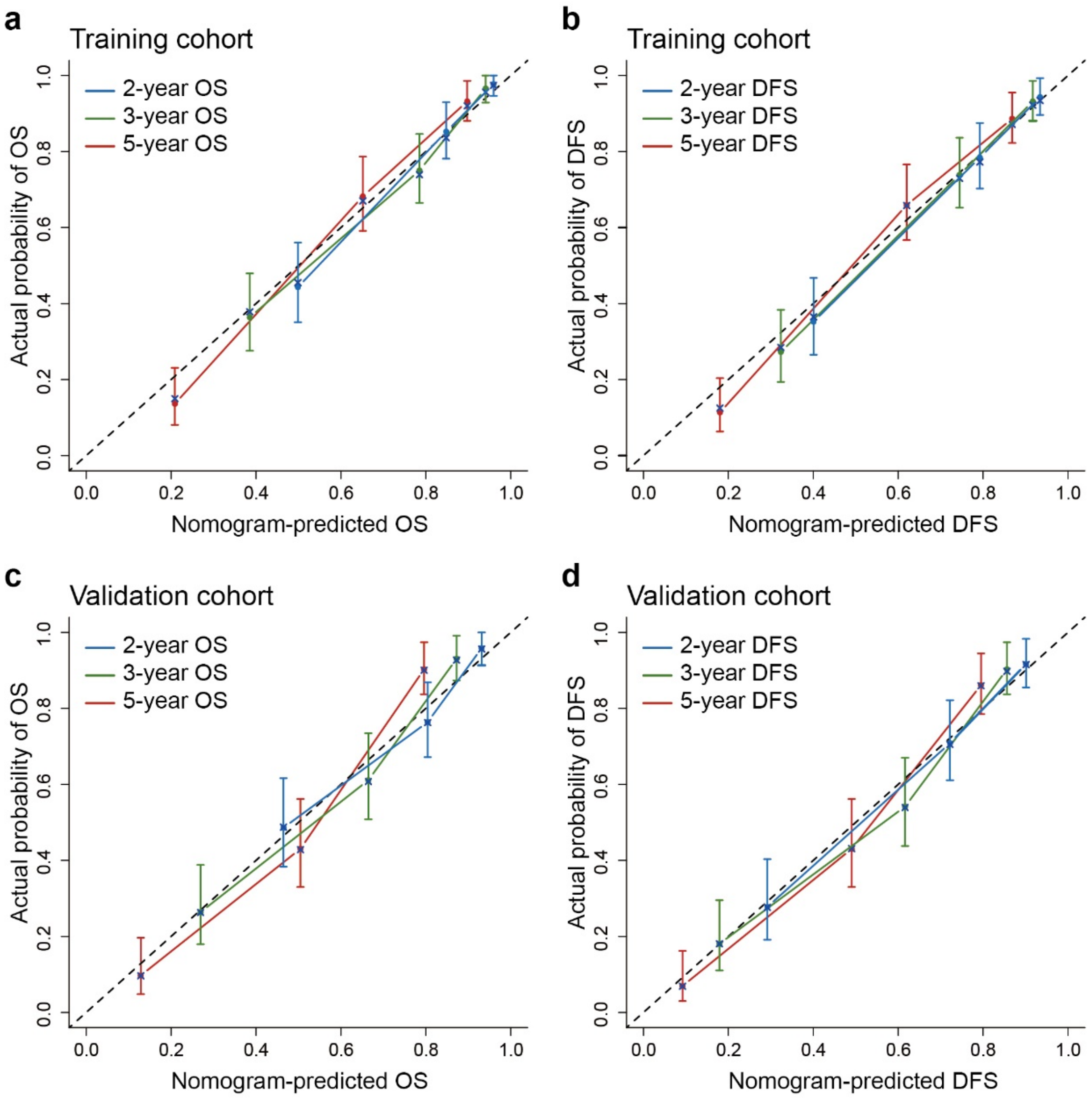

Supplementary Fig. 16. The calibration curves of the pathomics nomogram. **(a)** The calibration curves of the pathomics nomogram for OS in the training cohort. **(b)** The calibration curves of the pathomics nomogram for DFS in the training cohort. **(c)** The calibration curves of the pathomics nomogram for OS in the validation cohort. **(d)** The calibration curves of the pathomics nomogram for DFS in the validation cohort. Solid vertical lines and error bars represent the mean agreement between nomogram-predicted survival and actual probability of survival and the corresponding 95% confidence interval, respectively. In training cohort, n=264 patients. In validation cohort, n=216 patients. OS, overall survival; DFS, disease-free survival; TNM, tumour-node-metastasis; PS_{GC}, pathomics signature of gastric cancer. Source data are provided as a Source data file.

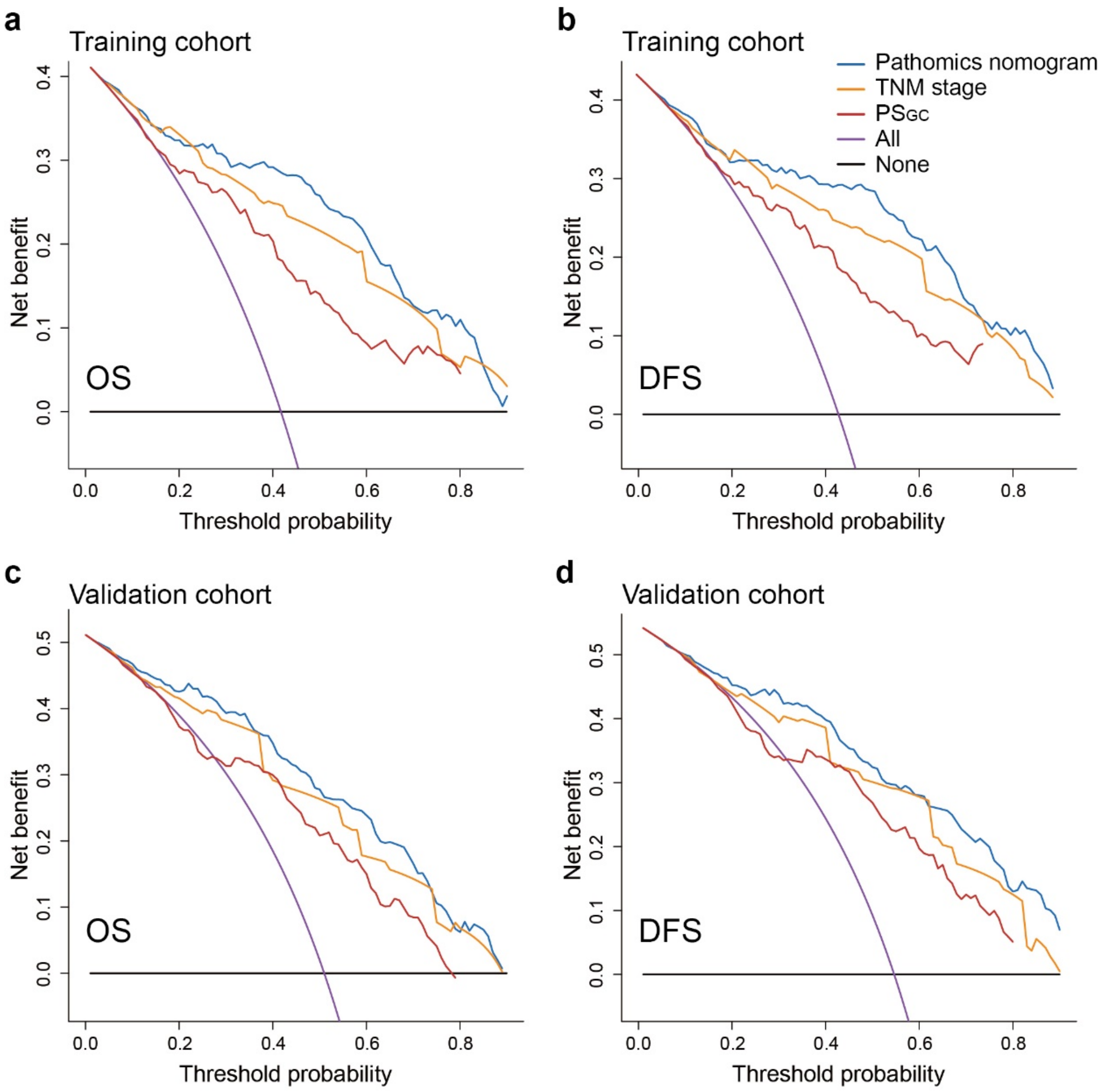

Supplementary Fig. 17. Decision curve analysis. **(a)** Decision curve analysis of OS for different models in the training cohort. **(b)** Decision curve analysis of DFS for different models in the training cohort. **(c)** Decision curve analysis of OS for different models in the validation cohort. **(d)** Decision curve analysis of DFS for different models in the validation cohort. In training cohort, n=264 patients. In validation cohort, n=216 patients. OS, overall survival; DFS, disease-free survival. Source data are provided as a Source data file.

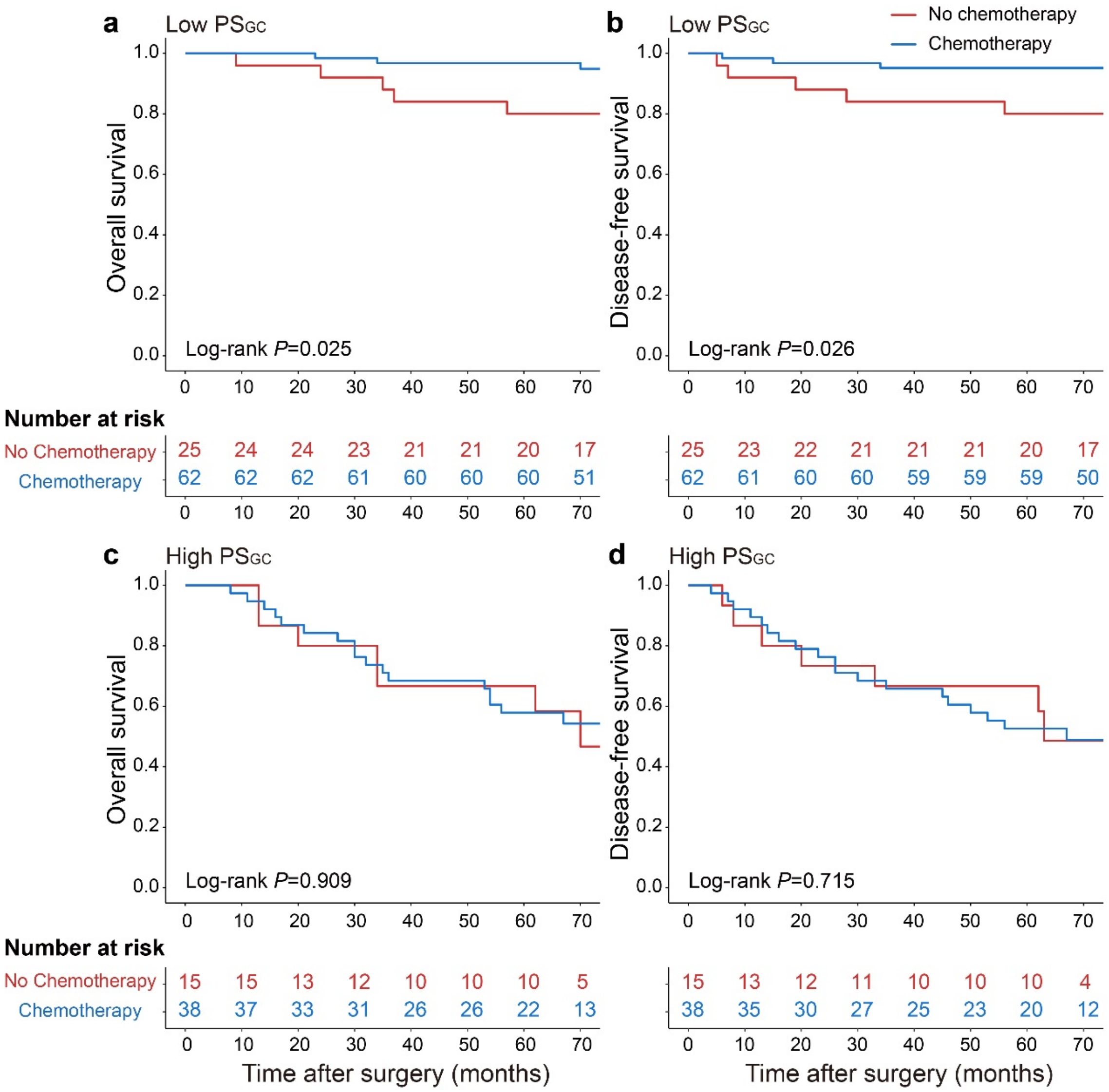

Supplementary Fig. 18. Adjuvant chemotherapy benefits for patients with stage II GC in terms of OS and DFS. **(a)** OS comparison according to the receipt of adjuvant chemotherapy in low-PS_{GC} stage II GC patients. **(b)** DFS comparison according to the receipt of adjuvant chemotherapy in low-PS_{GC} stage II GC patients. **(c)** OS comparison according to the receipt of adjuvant chemotherapy in high-PS_{GC} stage II GC patients. **(d)** DFS comparison according to the receipt of adjuvant chemotherapy in high-PS_{GC} stage II GC patients. The comparisons of OS and DFS between two groups are performed using a two-sided log-rank test. PS_{GC}, pathomics signature of gastric cancer; GC, gastric cancer; OS, overall survival; DFS, disease-free survival. Source data are provided as a Source data file.

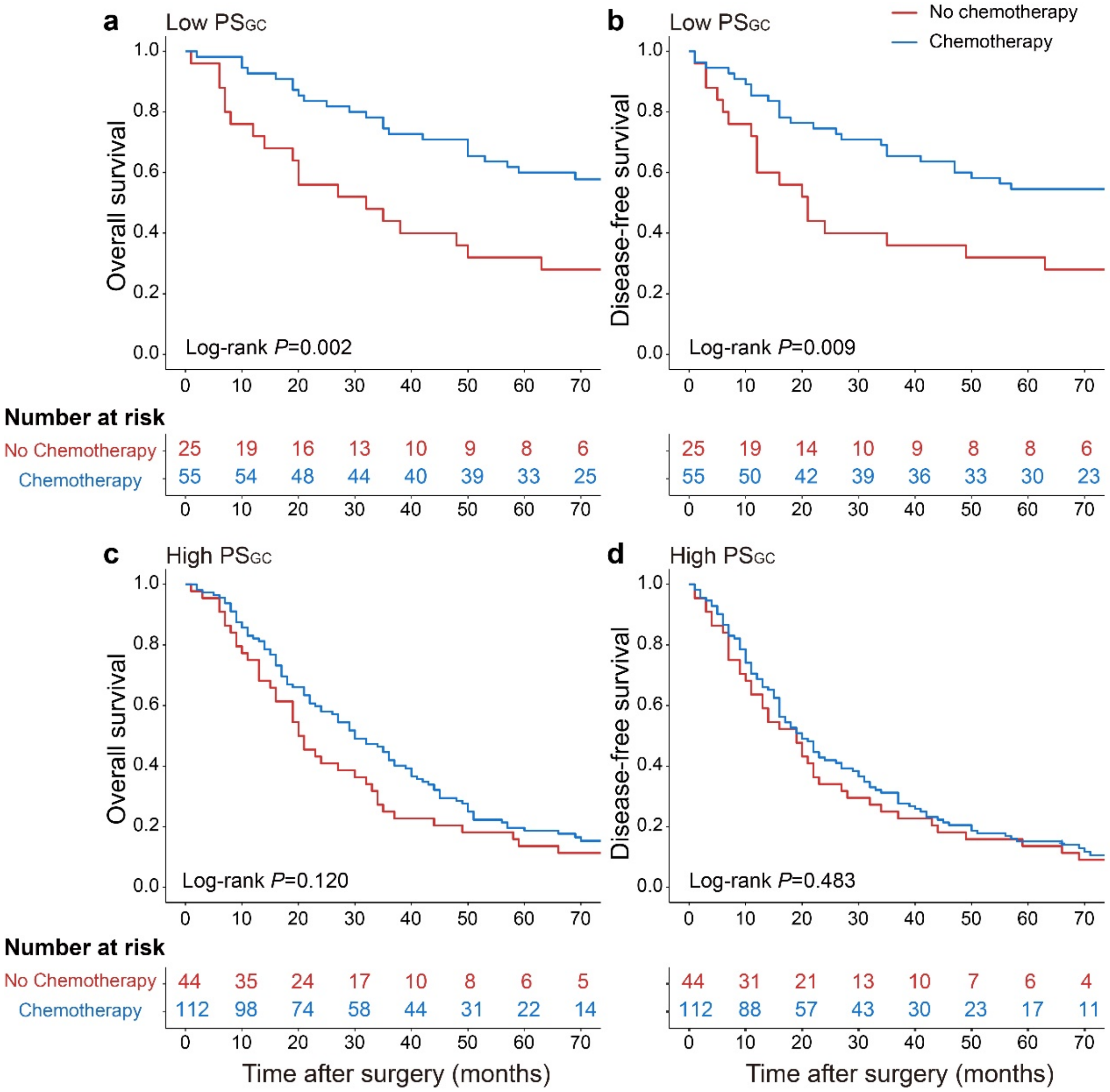

Supplementary Fig. 19. Adjuvant chemotherapy benefits for patients with stage III GC in terms of OS and DFS. **(a)** OS comparison according to the receipt of adjuvant chemotherapy in low-PS_{GC} stage III GC patients. **(b)** DFS comparison according to the receipt of adjuvant chemotherapy in low-PS_{GC} stage III GC patients. **(c)** OS comparison according to the receipt of adjuvant chemotherapy in high-PS_{GC} stage III GC patients. **(d)** DFS comparison according to the receipt of adjuvant chemotherapy in high-PS_{GC} stage III GC patients. The comparisons of OS and DFS between two groups are performed using a two-sided log-rank test. PS_{GC}, pathomics signature of gastric cancer; GC, gastric cancer; OS, overall survival; DFS, disease-free survival. Source data are provided as a Source data file.

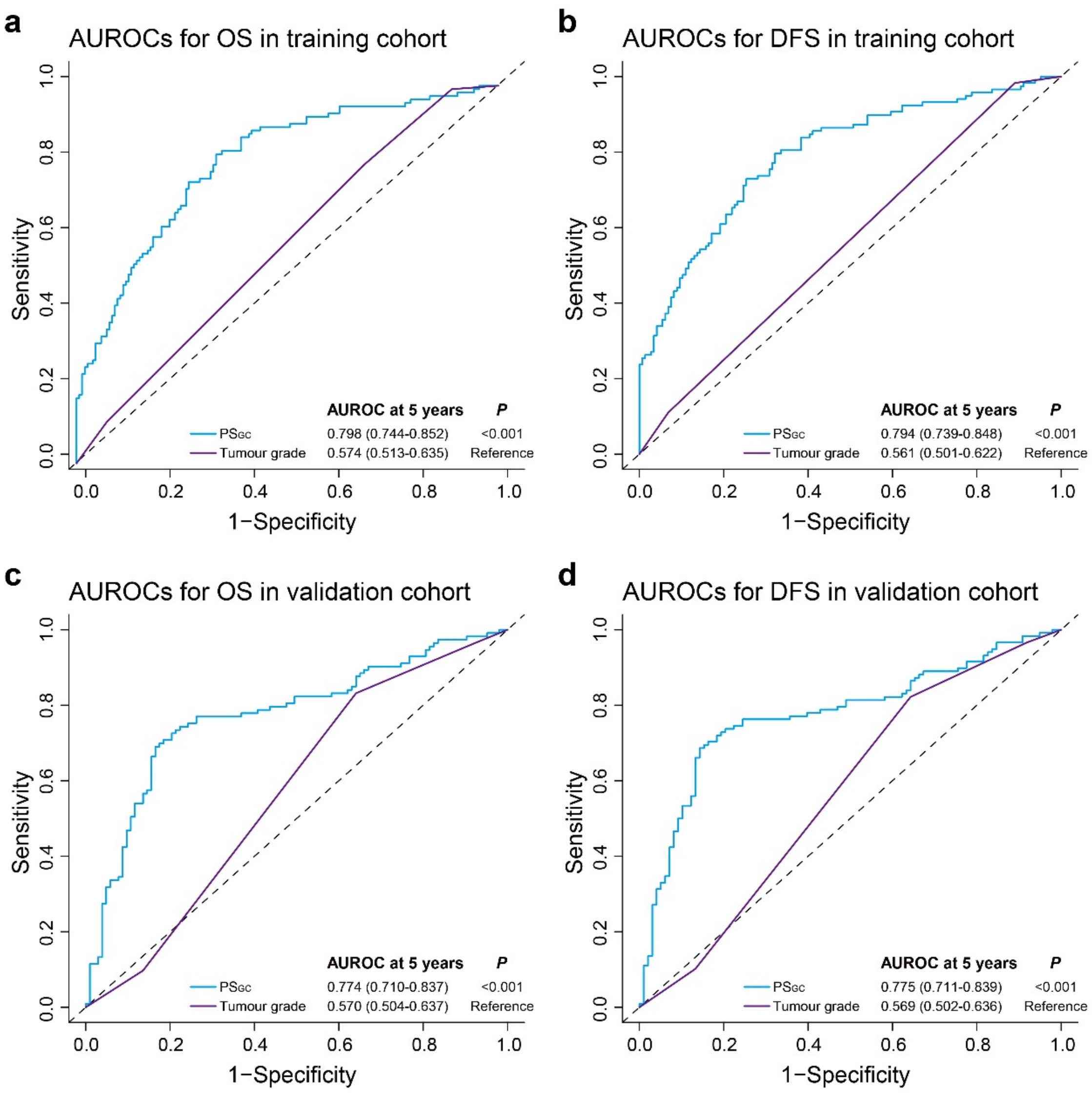

Supplementary Fig. 20. Comparison of AUROCs between the PS_{GC} and tumour grade. **(a)** Comparison of AUROCs for OS between the PS_{GC} and tumour grade in the training cohort. **(b)** Comparison of AUROCs for DFS between the PS_{GC} and tumour grade in the training cohort. **(c)** Comparison of AUROCs for OS between the PS_{GC} and tumour grade in the validation cohort. **(d)** Comparison of AUROCs for DFS between the PS_{GC} and tumour grade in the validation cohort. In training cohort, n=264 patients. In validation cohort, n=216 patients. The comparisons of AUROCs between two models are performed using a two-sided Delong test. AUROC, area under the receiver operating characteristic curve; OS, overall survival; DFS, disease-free survival; PS_{GC}, pathomics signature of gastric cancer. Source data are provided as a Source data file.

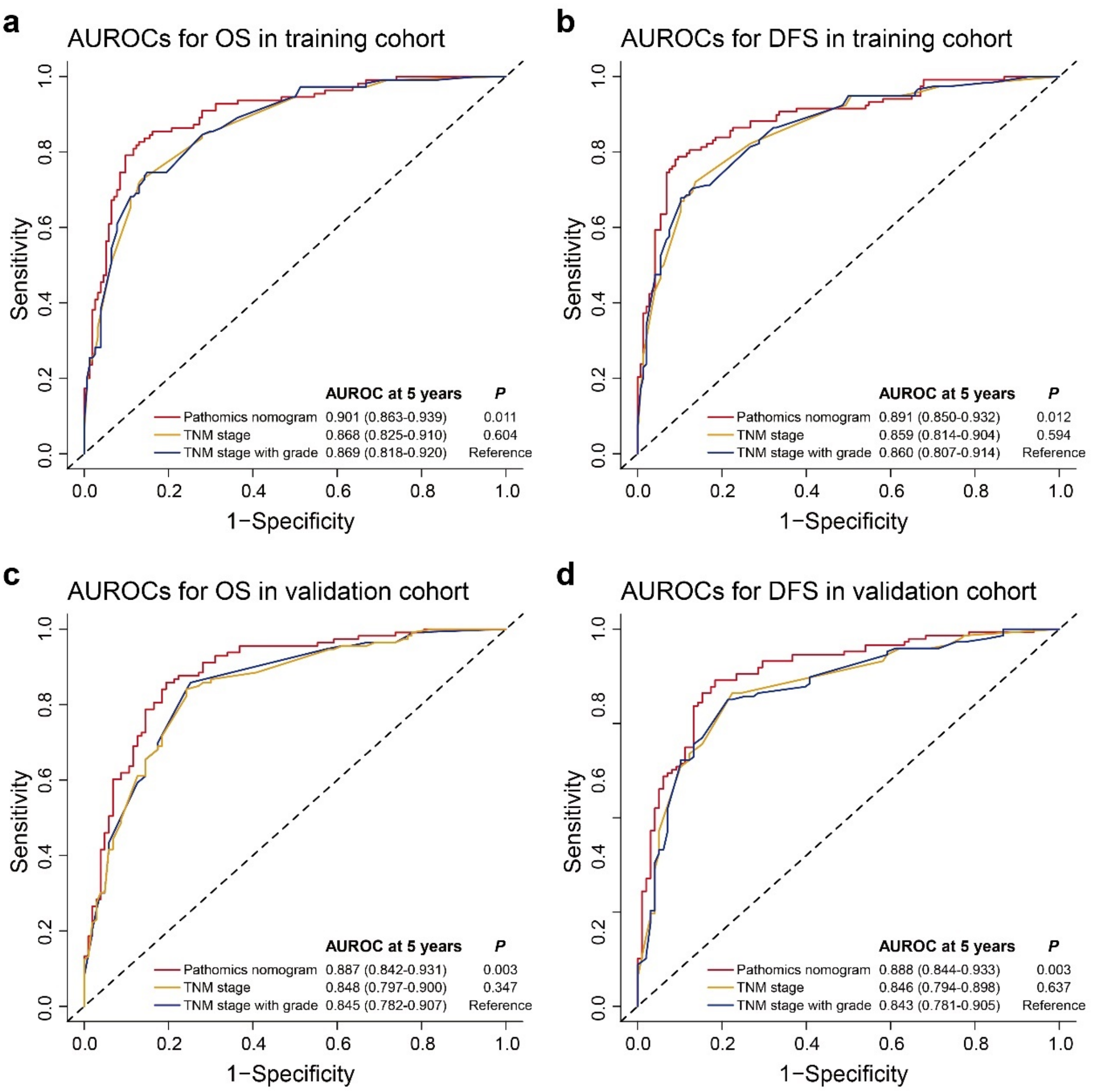

Supplementary Fig. 21. Comparison of AUROCs between the PS_{GC} and tumour grade when added to the TNM staging system. **(a)** Comparison of AUROCs for OS between the PS_{GC} and tumour grade when added to the TNM staging system in the training cohort. **(b)** Comparison of AUROCs for DFS between the PS_{GC} and tumour grade when added to the TNM staging system in the training cohort. **(c)** Comparison of AUROCs for OS between the PS_{GC} and tumour grade when added to the TNM staging system in the validation cohort. **(d)** Comparison of AUROCs for DFS between the PS_{GC} and tumour grade when added to the TNM staging system in the validation cohort. In training cohort, n=264 patients. In validation cohort, n=216 patients. The comparisons of AUROCs between two models are performed using a two-sided Delong test. AUROC, area under the receiver operating characteristic curve; OS, overall survival; DFS, disease-free survival; PS_{GC}, pathomics signature of gastric cancer; TNM, tumour-node-metastasis. Source data are provided as a Source data file.

Supplementary Note

$$
\begin{aligned}
PS_{GC} = &-2.063035280 \times \text{Correlation\_RWC\_Haematoxylin\_Eosin} \\
&+0.078385636 \times \text{Granularity\_H\&E\_11} \\
&-0.073252770 \times \text{Granularity\_Haematoxylin\_12} \\
&-0.003034897 \times \text{Granularity\_H\&E\_14} \\
&+0.066500543 \times \text{Granularity\_Haematoxylin\_14} \\
&-0.009349483 \times \text{Granularity\_Eosin\_15} \\
&-0.218289983 \times \text{Granularity\_H\&E\_5} \\
&-0.038121536 \times \text{Granularity\_Eosin\_6} \\
&+0.084302603 \times \text{Granularity\_Haematoxylin\_7} \\
&-0.002226299 \times \text{Granularity\_Eosin\_8} \\
&+0.194997260 \times \text{LocalFocusScore\_H\&E} \\
&+0.848055697 \times \text{PowerLogLogSlope\_H\&E} \\
&+3.2
\end{aligned}
$$

Supplementary Tables

Supplementary Table 1. Comparison of clinicopathological characteristics between patients with and without complete data

Variables	Complete data group (n=480)		Incomplete data group (n=24)		*P*
	n	%	n	%	
**Age**					0.453
≤ 60 years	283	58.9	16	66.7	
> 60 years	197	41.4	8	33.3	
**Age (years), median (IQR)**	58 (49-65)		55.5 (44-61.75)		0.120
**Sex**					0.880
Male	333	69.4	17	70.8	
Female	147	30.6	7	29.2	
**ECOG PS**					0.531
0	348	72.5	17	70.8	
1	123	25.6	6	25.0	
2	9	1.9	1	4.2	
**Tumour location**					0.999
Fundus of stomach	104	21.7	5	20.8	
Body of stomach	92	19.2	4	16.7	
Antrum of stomach	284	59.1	15	62.5	
**Tumour grade**					0.670
Grade 1	30	6.3	2	8.3	
Grade 2	98	20.4	6	25.0	
Grade 3	304	63.3	13	54.2	
Grade 4	48	10.0	3	12.5	
**Depth of invasion**					0.781
T1	78	16.3	4	16.7	
T2	41	8.5	2	8.3	
T3	48	10.0	4	16.7	
T4a	267	55.6	13	54.2	
T4b	46	9.6	1	4.1	
**Lymph node metastasis**					0.356
N0	193	40.2	14	58.3	
N1	90	18.6	4	16.7	
N2	80	16.7	4	16.7	
N3a	71	14.9	2	8.3	
N3b	46	9.6	0	0	
**Distant metastasis**					0.615
M0	460	95.8	24	100.0	
M1	20	4.2	0	0	

The comparison of continuous age variable between two groups are performed using a two-sided Mann-Whitney *U* test, and the rest variables are compared using a two-sided χ^2 or Fisher's exact test.

IQR, interquartile range; ECOG PS, Eastern Cooperative Oncology Group performance status.

Supplementary Table 2. Assessment of interaction effects between the PS_{GC} and depth of invasion

Variables	Overall survival		Disease-free survival	
	HR (95% CI)	*P*	HR (95% CI)	*P*
**PS_{GC}**	3.407 (0.237-48.877)	0.367	4.928 (0.619-39.231)	0.132
**Depth of invasion**		0.087		0.142
T1	Reference	-	Reference	-
T2	1.069 (0.018-61.327)	0.974	1.293 (0.038-43.960)	0.886
T3	2.152 (0.055-83.928)	0.682	2.491 (0.104-59.633)	0.573
T4a	8.041 (0.301-214.923)	0.214	7.912 (0.528-118.526)	0.134
T4b	21.979 (0.672-718.523)	0.082	17.223 (0.869-340.961)	0.062
**Lymph node metastasis**		<0.001		<0.001
N0	Reference	-	Reference	-
N1	1.462 (0.769-2.781)	0.247	1.263 (0.685-2.332)	0.453
N2	2.588 (1.391-4.814)	0.003	2.276 (1.261-4.107)	0.006
N3a	3.964 (2.163-7.264)	<0.001	3.896 (2.191-6.927)	<0.001
N3b	5.994 (3.023-11.884)	<0.001	4.703 (2.422-9.132)	<0.001
**Distant metastasis** (M1 vs. M0)	2.641 (1.362-5.119)	0.004	2.510 (1.301-4.839)	0.005
**PS_{GC}×Depth of invasion**		0.481		0.583
PS_{GC}×T1	Reference	-	Reference	-
PS_{GC}×T2	1.719 (0.074-39.917)	0.735	0.999 (0.073-13.634)	0.999
PS_{GC}×T3	1.173 (0.066-20.775)	0.913	0.824 (0.077-8.762)	0.872
PS_{GC}×T4a	0.624 (0.041-9.282)	0.732	0.458 (0.055-3.815)	0.470
PS_{GC}×T4b	0.449 (0.028-7.181)	0.571	0.342 (0.037-3.177)	0.346

Association of all variables with prognosis is analyzed using a two-sided Cox proportional hazard regression analysis.
PS_{GC}, pathomics signature of gastric cancer; HR, hazard ratio; CI, confidence interval.

Supplementary Table 3. Assessment of interaction effects between the PS_{GC} and lymph node metastasis

Variables	Overall survival		Disease-free survival	
	HR (95% CI)	*P*	HR (95% CI)	*P*
**PS_{GC}**	2.569 (1.024-6.444)	0.044	3.601 (1.510-8.592)	0.004
**Depth of invasion**		0.007		0.039
T1	Reference	-	Reference	-
T2	2.162 (0.498-9.377)	0.303	1.324 (0.372-4.711)	0.665
T3	2.586 (0.677-9.744)	0.166	1.947 (0.657-5.771)	0.230
T4a	4.591 (1.365-15.440)	0.014	3.007 (1.149-7.867)	0.025
T4b	7.645 (2.042-28.614)	0.003	4.075 (1.364-12.173)	0.012
**Lymph node metastasis**		<0.001		<0.001
N0	Reference	-	Reference	-
N1	0.192 (0.020-1.806)	0.147	0.419 (0.051-3.453)	0.419
N2	3.723 (0.672-20.666)	0.132	4.252 (0.806-22.432)	0.088
N3a	5.943 (1.003-35.222)	0.050	7.041 (1.183-41.919)	0.032
N3b	25.708 (4.035-163.789)	<0.001	52.349 (8.032-341.183)	<0.001
**Distant metastasis** (M1 vs. M0)	2.733 (1.378-5.421)	0.004	2.516 (1.282-4.936)	0.007
**PS_{GC}×Lymph node metastasis**		0.202		0.108
PS_{GC}×N0	Reference	-	Reference	-
PS_{GC}×N1	3.927 (0.954-16.168)	0.058	2.077 (0.543-7.953)	0.286
PS_{GC}×N2	0.755 (0.242-2.357)	0.629	0.621 (0.209-1.846)	0.391
PS_{GC}×N3a	0.738 (0.229-2.379)	0.610	0.642 (0.201-2.042)	0.452
PS_{GC}×N3b	0.434 (0.133-1.415)	0.166	0.241 (0.074-0.778)	0.017

Association of all variables with prognosis is analyzed using a two-sided Cox proportional hazard regression analysis. PS_{GC}, pathomics signature of gastric cancer; HR, hazard ratio; CI, confidence interval.

Supplementary Table 4. Assessment of interaction effects between the PS_{GC} and distant metastasis

Variables	Overall survival		Disease-free survival	
	**HR (95% CI)**	***P***	**HR (95% CI)**	***P***
**PS_{GC}**	2.263 (1.561-3.282)	<0.001	2.386 (1.642-3.469)	<0.001
**Depth of invasion**		0.018		0.047
T1	Reference	-	Reference	-
T2	2.289 (0.536-9.772)	0.875	1.440 (0.409-5.064)	0.569
T3	3.253 (0.878-12.046)	0.569	2.409 (0.826-7.030)	0.107
T4a	4.742 (1.431-15.715)	0.134	3.201 (1.233-8.304)	0.016
T4b	7.360 (1.987-27.257)	0.062	4.235 (1.428-12.564)	0.009
**Lymph node metastasis**		<0.001		<0.001
N0	Reference	-	Reference	-
N1	1.486 (0.769-2.872)	0.454	1.340 (0.719-2.496)	0.356
N2	2.502 (1.341-4.671)	0.007	2.268 (1.253-4.105)	0.006
N3a	3.776 (2.028-7.030)	<0.001	3.905 (2.163-7.051)	<0.001
N3b	5.939 (3.005-11.738)	<0.001	4.820 (2.485-9.350)	<0.001
**Distant metastasis** (M1 vs. M0)	5.313 (0.292-96.656)	0.259	2.798 (0.138-56.596)	0.502
**PS_{GC}×Distant metastasis**	0.641 (0.103-3.988)	0.633	0.942 (0.141-6.315)	0.951

Association of all variables with prognosis is analyzed using a two-sided Cox proportional hazard regression analysis.
PS_{GC}, pathomics signature of gastric cancer; HR, hazard ratio; CI, confidence interval.

Supplementary Table 5. Multivariate Cox regression analyses of overall survival and disease-free survival without the PS_{GC}

Variables	Overall survival		Disease-free survival	
	**HR (95% CI)**	***P***	**HR (95% CI)**	***P***
**Depth of invasion**		<0.001		0.002
T1	Reference	-	Reference	-
T2	2.328 (0.544-9.972)	0.255	1.492 (0.423-5.269)	0.534
T3	3.475 (0.936-12.902)	0.063	2.723 (0.933-7.943)	0.067
T4a	6.017 (1.825-19.837)	0.003	4.148 (1.610-10.688)	0.003
T4b	10.682 (2.943-38.767)	<0.001	6.070 (2.086-17.664)	0.001
**Lymph node metastasis**		<0.001		<0.001
N0	Reference	-	Reference	-
N1	1.582 (0.832-3.008)	0.162	1.432 (0.777-2.640)	0.250
N2	2.916 (1.580-5.381)	0.001	2.679 (1.497-4.794)	0.001
N3a	4.531 (2.486-8.260)	<0.001	4.684 (2.642-8.307)	<0.001
N3b	8.762 (4.615-16.636)	<0.001	7.600 (4.101-14.084)	<0.001
**Distant metastasis** (M1 vs. M0)	2.862 (1.475-5.551)	0.002	2.638 (1.363-5.106)	0.004

Association of all variables with prognosis is analyzed using a two-sided Cox proportional hazard regression analysis.
PS_{GC}, pathomics signature of gastric cancer; HR, hazard ratio; CI, confidence interval.

Supplementary Table 6. C-indexes for overall survival and disease-free survival in different models

Model	Training cohort		Validation cohort	
	**C-index (95% CI)**	***P***	**C-index (95% CI)**	***P***
**Overall survival**				
Pathomics nomogram	0.809 (0.741-0.878)	0.002	0.784 (0.706-0.862)	<0.001
TNM stage	0.782 (0.709-0.855)	Reference	0.742 (0.656-0.828)	Reference
PS_{GC}	0.727 (0.641-0.813)	-	0.725 (0.627-0.823)	-
**Disease-free survival**				
Pathomics nomogram	0.792 (0.718-0.866)	0.022	0.794 (0.709-0.873)	<0.001
TNM stage	0.770 (0.694-0.846)	Reference	0.748 (0.660-0.836)	Reference
PS_{GC}	0.712 (0.622-0.802)	-	0.738 (0.642-0.834)	-

The comparisons of C-indexes between two models are performed using a two-sided *z*-score test.

TNM, tumour -node-metastasis; PS_{GC}, pathomics signature of gastric cancer; CI, confidence interval.

Supplementary Table 7. Net reclassification improvement of adding the PS_{GC} to the TNM stage model

Model	Overall survival		Disease-free survival	
	NRI (95% CI)	*P*	NRI (95% CI)	*P*
**Pathomics nomogram vs. TNM stage**				
Training cohort	0.177 (0.021-0.319)	0.026	0.218 (0.048-0.344)	0.012
Validation cohort	0.318 (0.147-0.497)	0.010	0.380 (0.141-0.556)	0.028

The comparisons of NRI between two models are performed using a two-sided *Z* test.

PS_{GC}, pathomics signature of gastric cancer; TNM, tumour-node-metastasis; NRI, net reclassification improvement; CI, confidence interval.

Supplementary Table 8. Clinicopathological characteristics of patients with stage II and III disease according to receipt of chemotherapy

Variables	Number	No chemotherapy (n=109)		Chemotherapy (n=267)	
		n	%	n	%
**Age**					
≤60 years	221	50	45.9	171	64.0
>60 years	155	59	54.1	96	36.0
**Sex**					
Male	262	73	67.0	189	70.8
Female	114	36	33.0	78	29.2
**ECOG PS**					
0	282	82	75.2	200	74.9
1	88	26	23.9	62	23.2
2	6	1	0.9	5	1.9
**CEA level**					
Normal	306	87	79.8	219	82.0
Elevated	70	22	20.2	48	18.0
**CA 19-9 level**					
Normal	305	85	78.0	220	82.4
Elevated	71	24	22.0	47	17.6
**Tumour location**					
Fundus of the stomach	87	27	24.8	60	22.5
Body of the stomach	79	25	22.9	54	20.2
Antrum of the stomach	210	57	53.3	153	57.3
**Tumour size**					
≤4 cm	176	42	38.5	134	50.2
>4 cm	200	67	61.5	133	49.8
**Tumour grade**					
Grade 1	10	3	2.8	7	2.6
Grade 2	73	27	24.8	46	17.2
Grade 3	256	69	63.3	187	70.1
Grade 4	37	10	9.1	27	10.1
**Lauren type**					
Intestinal type	155	48	44.0	107	40.1
Diffuse and mixed type	221	61	56.0	160	59.9
**Depth of invasion**					
T1	8	2	1.8	6	2.2
T2	27	10	9.2	17	6.4
T3	47	13	11.9	34	12.7
T4a	252	72	66.1	180	67.4
T4b	42	12	11.0	30	11.3
**Lymph node metastasis**					
N0	111	31	28.4	80	30.0
N1	81	27	24.8	54	20.2

N2	78	31	28.4	47	17.6
N3a	64	15	13.8	49	18.4
N3b	42	5	4.6	37	13.8
**TNM stage**					
Stage II	140	40	36.7	100	37.5
Stage III	236	69	63.3	167	62.5

CEA, carcinoembryonic antigen; CA, carbohydrate antigen; TNM, tumour-node-metastasis; ECOG PS, Eastern Cooperative Oncology Group performance status.

Supplementary Table 9. Comparison of ECOG PS with receipt of adjuvant chemotherapy in patients with a high PS_{GC} and stage II or III diseases

**ECOG PS**	**Training cohort**					**Validation cohort**				
	**Chemotherapy**		**No chemotherapy**		***P***	**Chemotherapy**		**No chemotherapy**		***P***
	**n**	**%**	**n**	**%**		**n**	**%**	**n**	**%**	
0	63	70.0	21	72.4		43	71.7	22	73.3	
1	25	27.8	8	27.6	0.999	16	26.7	7	23.3	0.999
2	2	2.2	0	0		1	1.6	1	3.4	

The comparisons of variables between two groups are performed using a two-sided Fisher's exact test.

ECOG PS, Eastern Cooperative Oncology Group performance status.

Supplementary Table 10. Association of the PS_{GC} with overall survival and disease-free survival in stage II and III patients receiving adjuvant chemotherapy

PS_{GC} level	Adjuvant chemotherapy		Overall survival			Disease-free survival		
	No chemo	Chemo	HR (95% CI)	*P*	$P_{interaction}$	HR (95% CI)	*P*	$P_{interaction}$
**Stage II (n=140)**								
High PS_{GC} (Chemo vs. No chemo)	15	38	1.049 (0.436-2.521)	0.915	<0.001	1.174 (0.494-2.786)	0.717	<0.001
Low PS_{GC} (Chemo vs. No chemo)	25	62	0.224 (0.054-0.983)	0.041		0.226 (0.054-0.946)	0.042	
**Stage III (n=236)**								
High PS_{GC} (Chemo vs. No chemo)	44	112	0.745 (0.512-1.083)	0.123	0.005	0.875 (0.606-1.264)	0.477	<0.001
Low PS_{GC} (Chemo vs. No chemo)	25	55	0.392 (0.211-0.729)	0.003		0.453 (0.247-0.834)	0.011	

Association of all variables with prognosis is analyzed using a two-sided Cox proportional hazard regression analysis.

PS_{GC}, pathomics signature of gastric cancer; HR, hazard ratio; CI, confidence interval; Chemo, chemotherapy.

Supplementary Table 11. Comparison of C-indexes between the PS_{GC} and tumour grade

**Variable**	**Training cohort**		**Validation cohort**	
	**C-index (95% CI)**	***P***	**C-index (95% CI)**	***P***
**Overall survival**				
PS_{GC}	0.727 (0.641-0.813)	<0.001	0.725 (0.627-0.823)	<0.001
Tumour grade	0.569 (0.483-0.655)	Reference	0.562 (0.472-0.652)	Reference
**Disease-free survival**				
PS_{GC}	0.712 (0.622-0.802)	<0.001	0.738 (0.642-0.834)	<0.001
Tumour grade	0.553 (0.465-0.641)	Reference	0.552 (0.462-0.642)	Reference

The comparisons of C-indexes between two models are performed using a two-sided *z*-score test.

PS_{GC}, pathomics signature of gastric cancer; CI, confidence interval.

Supplementary Table 12. Comparison of C-indexes between the tumour grade and PS_{GC} when added to the TNM staging system

Model	Training cohort		Validation cohort	
	**C-index (95% CI)**	***P***	**C-index (95% CI)**	***P***
**Overall survival**				
Pathomics nomogram	0.809 (0.741-0.878)	0.008	0.784 (0.706-0.862)	<0.001
TNM stage	0.782 (0.709-0.855)	0.582	0.742 (0.656-0.828)	0.979
TNM stage combined with the tumour grade	0.784 (0.711-0.857)	Reference	0.743 (0.657-0.829)	Reference
**Disease-free survival**				
Pathomics nomogram	0.792 (0.718-0.866)	0.039	0.794 (0.709-0.873)	<0.001
TNM stage	0.770 (0.694-0.846)	0.675	0.748 (0.660-0.836)	0.318
TNM stage combined with the tumour grade	0.771 (0.695-0.847)	Reference	0.744 (0.654-0.834)	Reference

The comparisons of C-indexes between two models are performed using a two-sided *z*-score test.

TNM, tumour-node-metastasis; PS_{GC}, pathomics signature of gastric cancer; CI, confidence interval.

Supplementary Table 13. Comparison of C-indexes between the PS_{GC} and lymph node metastasis

Variable	Training cohort		Validation cohort	
	**C-index (95% CI)**	***P***	**C-index (95% CI)**	***P***
**Overall survival**				
PS_{GC}	0.727 (0.641-0.813)	0.708	0.725 (0.627-0.823)	0.822
Lymph node metastasis	0.737 (0.647-0.821)	Reference	0.717 (0.629-0.805)	Reference
**Disease-free survival**				
PS_{GC}	0.712 (0.622-0.802)	0.584	0.738 (0.642-0.834)	0.611
Lymph node metastasis	0.736 (0.646-0.820)	Reference	0.721 (0.631-0.811)	Reference

The comparisons of C-indexes between two variables are performed using a two-sided *z*-score test.
PS_{GC}, pathomics signature of gastric cancer; CI, confidence interval.

Supplementary Table 14. Comparison of AUROCs between the PS_{GC} and lymph node metastasis

Variable	Training cohort		Validation cohort	
	**AUROC (95% CI)**	***P***	**AUROC (95% CI)**	***P***
**Overall survival**				
PS_{GC}	0.798 (0.744-0.852)	0.575	0.774 (0.710-0.837)	0.354
Lymph node metastasis	0.796 (0.742-0.850)	Reference	0.809 (0.753-0.866)	Reference
**Disease-free survival**				
PS_{GC}	0.794 (0.739-0.848)	0.638	0.775 (0.711-0.839)	0.378
Lymph node metastasis	0.794 (0.741-0.847)	Reference	0.816 (0.760-0.872)	Reference

The comparisons of AUROCs between two variables are performed using a two-sided Delong test.

PS_{GC}, pathomics signature of gastric cancer; AUROC, area under the receiver operating characteristic curve; CI, confidence interval.

Supplementary Table 15. Summary of the pathomics features

Features classification	Features description
**Image quality features**	FocusScore_α
	LocalFocusScore_α
	Correlation_α
	PowerLogLogSlope_α
	MeanIntensity_α
	MedianIntensity_α
	StdIntensity_α
	MADIntensity_α
	LowerQuartileIntensity_α
	UpperQuartileIntensity_α
	Threshold_α
**Image colocalization features**	Correlation_Eosin_Haematoxylin
	Costes_Eosin_Haematoxylin
	Costes_Haematoxylin_Eosin
	Manders_Eosin_Haematoxylin
	Manders_Haematoxylin_Eosin
	Overlap_Eosin_Haematoxylin
	RWC_Eosin_Haematoxylin
	RWC_Haematoxylin_Eosin
	Slope_Eosin_Haematoxylin
**Image granularity features**	Granularity_α_β

α represents the type of images, which could be haematoxylin, eosin, and H&E;

β represents the granular spectrum, which could be 1, 2, 3, 4, ···, 15, 16.

H&E, haematoxylin and eosin.

Supplementary References

1. Carpenter, A.E. et al. CellProfiler: image analysis software for identifying and quantifying cell phenotypes. *Genome Biol.* **7**, R100 (2006).

2. Kamentsky, L. et al. Improved structure, function and compatibility for CellProfiler: modular high-throughput image analysis software. *Bioinformatics* **27**, 1179-80 (2011).

3. Ruifrok, A.C. & Johnston, D.A. Quantification of histochemical staining by color deconvolution. *Anal. Quant. Cytol. Histol.* **23**, 291-9 (2001).

4. Bray, M.A., Fraser, A.N., Hasaka, T.P. & Carpenter, A.E. Workflow and metrics for image quality control in large-scale high-content screens. *J. Biomol. Screen.* **17**, 266-74 (2012).

5. Field, D.J. Relations between the statistics of natural images and the response properties of cortical cells. *J. Opt. Soc. Am. A* **4**, 2379-94 (1987).

6. Haralick, R.M. Statistical and structural approaches to texture. *Proc. IEEE* **67**, 786-804 (1979).

7. Sun, Y., Duthaler, S. & Nelson, B.J. Autofocusing in computer microscopy: selecting the optimal focus algorithm. *Microsc. Res. Tech.* **65**, 139-49 (2004).

8. Otsu, N. A thresholding selection method from gray-level histogram. *IEEE Trans. Syst. Man. Cybern.* **9**, 62-66 (1979).

9. Aaron, J.S., Taylor, A.B. & Chew, T.L. Image co-localization - co-occurrence versus correlation. *J. Cell. Sci.* **131**(2018).

10. Costes, S.V. et al. Automatic and quantitative measurement of protein-protein colocalization in live cells. *Biophys J.* **86**, 3993-4003 (2004).

11. Singan, V.R., Jones, T.R., Curran, K.M. & Simpson, J.C. Dual channel rank-based intensity weighting for quantitative co-localization of microscopy images. *BMC Bioinformatics* **12**, 407 (2011).

12. Vincent, L. Granulometries and opening trees. *Fund. Inform.* **41**, 57-90 (2000).

13. Maragos, P. Pattern spectrum and multiscale shape representation. *IEEE Trans. Pattern. Anal. Mach. Intell.* **11**, 701 - 716 (1989).
