# Supplementary material for: Prognostic and predictive value of a pathomics signature in gastric cancer
Source: Nat Commun. 2022 Nov 12;13:6903. doi: 10.1038/s41467-022-34703-w (PMC9653436; doi:10.1038/s41467-022-34703-w)
Supplement: Supplementary file 2 — Reporting Summary [file 41467_2022_34703_MOESM2_ESM.pdf]

nature portfolio

Corresponding author(s): Jun Yan
Last updated by author(s): Nov 2, 2022

Reporting Summary

Nature Portfolio wishes to improve the reproducibility of the work that we publish. This form provides structure for consistency and transparency in reporting. For further information on Nature Portfolio policies, see our Editorial Policies and the Editorial Policy Checklist.

Statistics

For all statistical analyses, confirm that the following items are present in the figure legend, table legend, main text, or Methods section.

n/a	Confirmed	
☐	☒	The exact sample size (*n*) for each experimental group/condition, given as a discrete number and unit of measurement
☐	☒	A statement on whether measurements were taken from distinct samples or whether the same sample was measured repeatedly
☐	☒	The statistical test(s) used AND whether they are one- or two-sided<br>*Only common tests should be described solely by name; describe more complex techniques in the Methods section.*
☐	☒	A description of all covariates tested
☐	☒	A description of any assumptions or corrections, such as tests of normality and adjustment for multiple comparisons
☐	☒	A full description of the statistical parameters including central tendency (e.g. means) or other basic estimates (e.g. regression coefficient) AND variation (e.g. standard deviation) or associated estimates of uncertainty (e.g. confidence intervals)
☐	☒	For null hypothesis testing, the test statistic (e.g. *F*, *t*, *r*) with confidence intervals, effect sizes, degrees of freedom and *P* value noted<br>*Give P values as exact values whenever suitable.*
☒	☐	For Bayesian analysis, information on the choice of priors and Markov chain Monte Carlo settings
☐	☒	For hierarchical and complex designs, identification of the appropriate level for tests and full reporting of outcomes
☐	☒	Estimates of effect sizes (e.g. Cohen's *d*, Pearson's *r*), indicating how they were calculated

Our web collection on statistics for biologists contains articles on many of the points above.

Software and code

Policy information about availability of computer code

Data collection	All H&E slides were scanned by using the Aperio ScanScope Scanner system (Leica Biosystems) with the ×20 objective, and images were digitized as svs. format files, which were managed with the Aperio ImageScope software (version 12.4.6).<br><br>The quantitative pathomics features of the selected tiles were extracted using CellProfiler (version 4.0.7), an open source image analysis software developed by the Broad Institute (Cambridge, MA), which can be downloaded from the https://cellprofiler.org/.
Data analysis	All statistical analyses were performed using R software (version 4.0.5) and SPSS software (version 19.0). The LASSO-Cox regression method was performed using the "glmnet" (version 4.1.1) package. The PH assumption was checked using the "CoxPhLb" (version 1.2.0) package. The development, validation and performance assessment of the prognostic nomograms were conducted using the "rms" (version 6.2.0) package. Comparisons of C-indexes between different models were performed using the "compareC" (version 1.3.2) package. The time-dependent ROC curves were plotted using "riskRegression" (version 2020.12.08) package. Decision curve analysis was performed with the function of "stdca.R". The "survminer" (version 0.4.9) package was used for computing survival analyses.<br><br>The codes used for the slide image process and feature extraction are publicly available at https://github.com/CellProfiler/CellProfiler. The codes used to develop and evaluate the prediction model are publicly available at https://github.com/Dexin-Chen/Pathomics-analysis.

For manuscripts utilizing custom algorithms or software that are central to the research but not yet described in published literature, software must be made available to editors and reviewers. We strongly encourage code deposition in a community repository (e.g. GitHub). See the Nature Portfolio guidelines for submitting code & software for further information.

Data

Policy information about availability of data

All manuscripts must include a data availability statement. This statement should provide the following information, where applicable:

- Accession codes, unique identifiers, or web links for publicly available datasets
- A description of any restrictions on data availability
- For clinical datasets or third party data, please ensure that the statement adheres to our policy

The H&E images and clinical information analyzed during the current study are not publicly available for patient privacy purposes. Data access can be obtained through a reasonable request to Jun Yan (yanjunfudan@163.com). Access to the data will be restricted to non-commercial researches which remove patient-sensitive information. All requests will be answered within 10 working days. The source data underlying Figs. 2-4 and Supplementary Figs. 1-21 are provided as a Source data file. Source data are provided with this paper.

Human research participants

Policy information about studies involving human research participants and Sex and Gender in Research.

Reporting on sex and gender	The term sex was used in this study. No findings apply to one sex, and the sex was considered in study design because both male and female would suffer from gastric cancer. Of the 480 patients included in this study, 69.4% (333/480) were male, 30.6% (147/480) were female. The sex information of each participant was provided in the source data file, and consent has been obtained for sharing of individual-level data. The difference of sex between the training and validation cohorts was compared, and no difference was found.
Population characteristics	Of the 480 patients included in this study, 69.4% (333/480) were male, and the median (IQR) age was 58 (49-65) years. The majority of patients (76.3%, 366/480) were diagnosed with stage II or III disease. No significant difference in clinicopathological characteristics between the training and validation cohorts was found. The median (IQR) follow-up duration in the training cohort was 64 (27-72) months, with 5-year OS and DFS rates of 58.7% and 55.3%, respectively. In the validation cohort, the median (IQR) follow-up duration was 55 (22-92) months. The 5-year OS and DFS rates were 47.7% and 45.4%, respectively.
Recruitment	A training cohort including 264 consecutive patients from March 2012 to December 2013 at Nanfang Hospital of Southern Medical University was built. The inclusion criteria were as follows: (i) histologically diagnosed GC with curative surgery; (ii) at least 15 lymph nodes harvested; (iii) no history of other malignancies; (iv) complete clinicopathological and follow-up information. Patients receiving neoadjuvant chemotherapy, radiotherapy or chemoradiotherapy were excluded. A total of 216 consecutive patients were enrolled from Fujian Provincial Cancer Hospital of Fujian Medical University between August 2010 and September 2012. In this retrospective study, all consecutive patients were included strictly according to the inclusion and exclusion criteria, and the clinicopathological characteristics between patients with and without complete data were similar (Supplementary Table 1), thus potential self selection bias or other biases were reduced markedly, which were unlikely to impact the results.
Ethics oversight	This study was approved by the Institutional Review Boards of Nanfang Hospital of Southern Medical University and the Fujian Cancer Hospital of Fujian Medical University. Written informed consent was obtained from all patients before surgery, which contained a statement on the formalin-fixed, paraffin-embedded samples and clinicopathological data for scientific research.

Note that full information on the approval of the study protocol must also be provided in the manuscript.

Field-specific reporting

Please select the one below that is the best fit for your research. If you are not sure, read the appropriate sections before making your selection.

☒ Life sciences ☐ Behavioural & social sciences ☐ Ecological, evolutionary & environmental sciences

For a reference copy of the document with all sections, see nature.com/documents/nr-reporting-summary-flat.pdf

Life sciences study design

All studies must disclose on these points even when the disclosure is negative.

Sample size	For developing a prognostic nomogram, consensus of sample-size calculation has not yet been reached. According to the TRIPOD Statement (Moons et al., 2015, Ann Intern Med), at least 10 outcome events per variable (EPV) was needed. However, as the Statement noted, the 10 EPV was based on two empirical investigations (Peduzzi et al., 1995, J Clin Epidemiol; Peduzzi et al., 1996, J Clin Epidemiol). Some researches suspected that the 10 EPV is too lenient (Wynants et al., 2015, J Clin Epiermiol), or too strict (Vittinghoff et al., 2007, Am J Epidemiol). Only for a planned prospective prediction model development study will the sample size be predetermined on statistical grounds (Karel et al., 2015, Ann Intern Med). In this study, a total of 4 variables were included to develop the pathomics nomograms. There were 122 patients suffered from recurrence after radical surgery (more than 40), which was acceptable. For sample size calculation in the validation cohort, a previous study, Lei et al reported that the ratio between training and validation was at least 7:3 (Lei et al., 2015, JAMA Surgery). In our study, the

validation cohort contains 216 consecutive patients, which was also adequate.

Data exclusions	Patients receiving neoadjuvant chemotherapy, radiotherapy or chemoradiotherapy were excluded because anticancer therapy before radical surgery could affect the oncological outcomes. The exclusion criteria were pre-established before conducting data collection and analysis.
Replication	After the development of the pathomics signature and prognostic nomograms based on the training cohort with 264 patients, the validation cohort with 216 independently patients was used to externally verify the reproducibility of the predictive performance of the pathomics signature and nomograms. All attempts at replication were successful.
Randomization	The aim of this study was to develop and validate a pathomics signature to predict prognosis and adjuvant chemotherapy benefits in gastric cancer. Both the training and validation cohorts were consecutive patients, therefore, randomization was not needed.
Blinding	Pathologists were blinded to clinical characteristics and prognosis of the gastric cancer patients when selecting the regions of interest for features extraction.

Reporting for specific materials, systems and methods

We require information from authors about some types of materials, experimental systems and methods used in many studies. Here, indicate whether each material, system or method listed is relevant to your study. If you are not sure if a list item applies to your research, read the appropriate section before selecting a response.

Materials & experimental systems

n/a	Involved in the study	
☒	☐	Antibodies
☒	☐	Eukaryotic cell lines
☒	☐	Palaeontology and archaeology
☒	☐	Animals and other organisms
☒	☐	Clinical data
☒	☐	Dual use research of concern

Methods

n/a	Involved in the study	
☒	☐	ChIP-seq
☒	☐	Flow cytometry
☒	☐	MRI-based neuroimaging
